# Copper/Copper Oxide Nanoparticles: Biological Synthesis, Characterization and Potential Biomedical Applications: Advances and Perspectives

**DOI:** 10.3390/ph19081306

**Published:** 2026-08-18

**Authors:** Md. Amdadul Huq, Md. Ashikur Rahman, Md. Rasel Rana, Jong-Whi Park

**Affiliations:** 1 Department of Life Sciences, College of BioNano Technology, Gachon University, Seongnam 13120, Republic of Korea; 2 Food Safety and Regulatory Science, Chung-Ang University, Anseong-si 17546, Republic of Korea; apu.m1989@gmail.com; 3 Bangladesh Fisheries Research Institute, Shrimp Research Station, Bagerhat 9300, Bangladesh; 4 Department of Microbiology, Faculty of Science and Engineering, Rabindra Maitree University, Kushtia 7000, Bangladesh; raselrana8659@gmail.com

**Keywords:** Cu/CuO-NPs, biological synthesis, characterization, antibacterial properties, antifungal properties, antiviral properties, anticancer properties

## Abstract

The biosynthesis of copper and copper oxide nanoparticles (Cu/CuO-NPs) has attracted considerable interest due to its non-toxic, eco-friendly nature and wide-ranging applications, especially in nanomedicine and biomedical fields. Traditional nanoparticle production methods often involve toxic chemicals and generate harmful byproducts. In contrast, biological synthesis provides a cleaner, safer, more cost-effective, and sustainable alternative. Various biological sources, including plants, bacteria, fungi, and yeast, have been employed for the efficient and non-toxic production of Cu/CuO-NPs. These organisms contain diverse biomolecules such as enzymes, proteins, amino acids, vitamins, flavonoids, and alkaloids that function as reducing, capping, and stabilizing agents during nanoparticle formation. The biologically synthesized Cu/CuO-NPs are characterized using UV-VIS spectroscopy, Raman spectroscopy, TEM, SEM, EDX, XRD, TGA, XPS, FTIR, DLS, zeta potential analyzer, etc. Cu/CuO-NPs hold promise for applications in nanomedicine, primarily because of their strong antimicrobial and anticancer activities and potential use as disinfectants against infectious diseases. Various reports have suggested that the biologically synthesized Cu/CuO-NPs have exhibited significant antimicrobial and anticancer efficacies against pathogenic bacteria, fungi and viruses and various cancer cells. Due to their nanoscale dimensions and extensive surface area, Cu/CuO nanoparticles can readily infiltrate cell walls, disrupt membrane integrity, generate reactive oxygen species, and hinder both DNA replication and protein production, leading to cell death. The present review comprehensively describes the biological synthesis of Cu/CuO-NPs, their characterization techniques, and potential antibacterial, antifungal, antiviral, and anticancer applications. The modes of action for antibacterial, antifungal, antiviral, and anticancer properties have also been explored critically.

## 1. Introduction

Nanomedicines are playing an increasingly vital role in healthcare by leveraging nanotechnology to enhance drug delivery, improve diagnostic capabilities, and develop novel therapeutic strategies. They offer targeted and controlled drug release, minimize side effects, and enable more effective treatments for various diseases [1,2,3,4]. Nanomedicine is a developing discipline that applies the principles and methods of nanoscience to medical biology for the prevention, diagnosis, and treatment of diseases. It involves the use of nanoscale materials such as nanorobots and nano-sensors for diagnostic, therapeutic, and sensing applications, as well as the activation of materials within living cells [1,2]. Nanoparticles are a key component of nanomedicine. Nanoparticles, with their unique properties at the nanoscale (typically 1–100 nm), play a crucial role in nanomedicine by enabling targeted drug delivery, enhanced imaging, and improved therapeutic outcomes [5,6,7]. They offer unique properties like tunable size, surface chemistry, and biocompatibility, allowing targeted drug delivery to particular tissues or cells while reducing side effects [8,9,10,11]. Among different nanoparticles, copper and copper oxide nanoparticles (Cu/CuO-NPs) have garnered significant attention for their diverse biomedical applications, including antimicrobial, anticancer, antioxidant, and anti-inflammatory roles. Their unique properties, such as high electrical conductivity, low cost, and ability to generate reactive oxygen species (ROS), contribute to their effectiveness in these areas [12,13,14,15].

Bioactive nanoparticles can be synthesized through chemical, physical, and biological methods [16,17,18]. Chemical methods involve the use of various chemicals and reducing agents, while physical methods utilize techniques like milling or laser ablation to reduce bulk materials into nanoparticles. Nonetheless, these methods come with several disadvantages, such as reliance on toxic reagents, complex purification processes, low conversion efficiency, inconsistent yields, environmental risks, significant energy demands, and elevated costs [17,19]. Biological methods leverage living organisms or their components to synthesize nanoparticles, often using plants, bacteria, or fungi [17,20,21]. Biological methods for nanoparticle synthesis can overcome some of the drawbacks associated with physical and chemical methods. Biological synthesis, often referred to as “green synthesis,” offers advantages like eco-friendliness, cost-effectiveness, and scalability, making it a promising alternative to traditional methods [22,23,24]. Moreover, nanoparticles synthesized using biological methods often show greater efficacy than chemically synthesized nanoparticles in various applications, particularly in drug delivery, anticancer and antimicrobial treatments. This is primarily due to their reduced toxicity, enhanced biocompatibility, and ability to be modified with targeting molecules [25,26]. Plant-based and microbe-mediated Cu/CuO-NPs exhibit commonalities in green synthesis approaches but differ in their characteristics and functional applications due to variations in synthesis pathways and the nature of stabilizing agents. Nanoparticles synthesized using phytochemicals derived from plants generally exhibit relatively larger particle dimensions and improved physicochemical stability. In contrast, microbial-mediated synthesis, especially when nanoparticles are produced through extracellular mechanisms, commonly results in smaller-sized particles that demonstrate comparatively greater biological activity. Both categories demonstrate broad applications, such as antimicrobial, anticancer, antioxidant, and catalytic roles; however, their distinct physicochemical traits determine their suitability for specific uses [27,28].

Pathogenic microorganisms pose a serious global health problem. They cause a wide range of illnesses, from mild to severe, and can even be fatal, leading to millions of deaths annually. Their impact extends beyond human health, affecting various sectors like agriculture, animal husbandry, and the environment [29,30]. They cause a vast array of diseases, including bacterial infections like pneumonia and urinary tract infections, viral infections like influenza and Ebola, fungal infections like histoplasmosis, and parasitic infections like malaria [29,31]. Outbreaks and infections can lead to social disruption, panic, and significant economic losses due to illness, treatment, and reduced productivity [32]. Increasing drug resistance in pathogenic microorganisms is a profoundly serious global health concern. It significantly limits treatment options for microbial infections, resulting in prolonged illness, increased medical expenses, and elevated death rates. The rise in antimicrobial resistance is a major threat that requires urgent attention and multifaceted interventions [33]. Cancer is a multifaceted and highly heterogeneous pathological condition in which abnormal cells undergo uncontrolled proliferation and acquire the capacity to invade and spread to distant sites within the body. Despite significant advancements in diagnostic methods, therapeutic strategies, and healthcare technologies, cancer remains one of the major causes of death globally. Conventional treatment modalities, such as chemotherapy, radiation therapy, and surgery, often face challenges, including limited drug delivery efficiency, severe adverse effects, and substantial financial burden. These challenges underscore the need for the development of more effective, targeted, and accessible therapeutic approaches for cancer management. Biosynthesized Cu/CuO-NPs may serve as promising antimicrobial and anticancer agents, due to their strong multi-mechanism action and eco-friendly production methods. Numerous studies indicate that Cu/CuO-NPs possess significant antimicrobial and anticancer properties [14,34,35,36,37]. These nanoparticles can damage microbial or cancer cells, leading to their inactivation, and are effective against various microorganisms, including bacteria, fungi and viruses and various type of cancers [14,34,38,39,40,41]. This review presents and discusses the biological synthesis of Cu/CuO-NPs, their characterization techniques and their advances, challenges and perspectives in the field of antibacterial, antifungal, antiviral and anticancer applications.

## 2. Biological Synthesis of Cu/CuO-NPs

**Figure 1:**
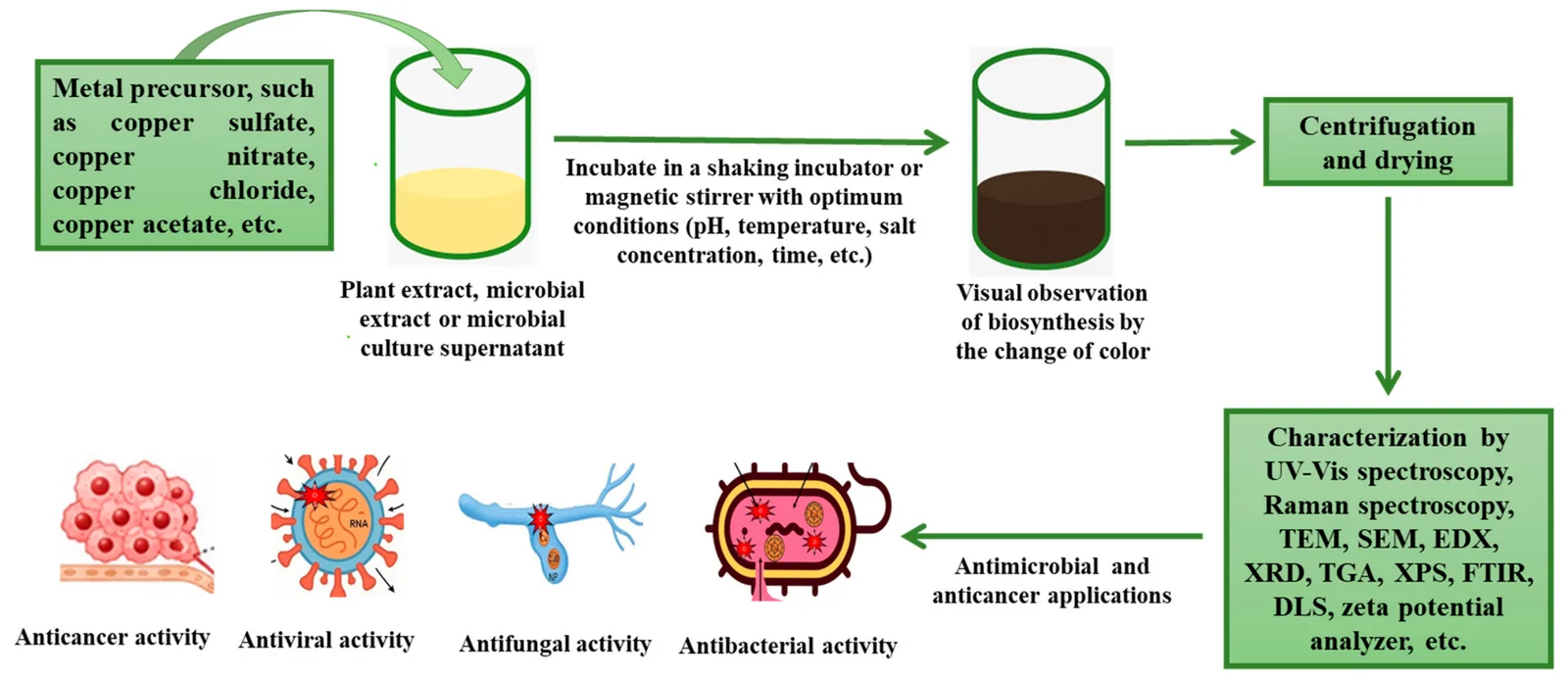
Schematic illustration of biological synthesis, characterization, and potential biomedical applications of Cu/CuO-NPs.

Biological synthesis of nanoparticles, also known as green synthesis, is a method that utilizes biological systems like plants, bacteria, fungi, and algae to produce nanoparticles. This approach is gaining popularity as a sustainable and eco-friendly alternative to traditional chemical and physical methods [16,17,42]. Biological synthesis relies on the presence of biomolecules like enzymes, proteins, polyphenols, and other reducing agents within the biological system to convert metal ions into nanoparticles [43]. Biological synthesis offers several advantages, including eco-friendliness, cost-effectiveness, and tunable properties [17,44,45]. Copper/copper oxide nanoparticle (Cu/CuO-NPs) biogenesis refers to the environmentally friendly process of creating materials by combining a solvent, a suitable reducing agent, and a safe stabilizing agent [46]. This method of synthesis produces stable chemicals and is simple, economical, dependable, sustainable, and repeatable. Copper ions can exist in several different oxidation states, including Cu(I), Cu(II), and Cu(III) [47]. The synthesis methods for CuO, Cu_2_O, and Cu_4_O_3_ are similar in terms of temperature, pH, precursor concentration, and the type of extract used, whether it be from plants, fungi, algae, or bacteria [48]. However, these factors significantly influence the specific type of copper particles produced during green synthesis. During this process, the Cu^2+^ ion is reduced to Cu^0^ while simultaneously oxidizing to form CuO nanoparticles, with the help of biomolecules in the extract. Some of these biomolecules stabilize the nanoparticles and act as capping agents [27]. The fundamental elements required for the biosynthesis of Cu/CuO-NPs are copper precursors and biological agents. Copper precursors typically consist of different copper salts, such as copper sulfate, copper nitrate, or copper chloride, which supply the necessary copper ions. Biological sources include plants, microorganisms, algae, and purified biomolecules [49,50]. Plant extracts—derived from leaves, peels, stems, seeds, or fruits—are rich in polyphenols, flavonoids, sugars, organic acids, and proteins, which serve both as reducing agents for Cu^2+^ and as stabilizers for the resulting nanoparticles [49]. Microbes such as bacteria, fungi, and yeast produce enzymes (e.g., nitrate reductase, reductive proteins), extracellular polysaccharides, and peptides that enable nanoparticle formation either within cells (intracellular) or outside cells through secreted reducing compounds (extracellular) [50]. Algal species contain pigments and bioactive metabolites capable of reducing metal ions and stabilizing nanoparticles, making them suitable for environmentally friendly synthesis. Additionally, purified biomolecules such as polyphenols, proteins, amino acids, and plant polysaccharides are also employed in nanoparticle biosynthesis. The biosynthesis mechanisms of Cu/CuO-NPs differ depending on the biological system used, yet they generally follow three main stages: reduction, nucleation and growth, and capping/stabilization [51,52]. During the reduction phase, biomolecules such as polyphenols, sugars, and enzymes present in plant or microbial extracts act as electron donors, converting Cu^2+^ ions (commonly derived from salts like CuSO_4_) into Cu^0^ or lower oxidation states. Under aqueous and aerobic conditions, the freshly reduced copper rapidly oxidizes to CuO or Cu_2_O unless protected by a controlled atmosphere or stabilized by strong capping agents [51]. In the nucleation and growth stage, reduced copper atoms aggregate to form nuclei, with the rate of growth influenced by factors such as reducing capacity, precursor concentration, temperature, and the presence of stabilizers. Finally, during the capping and stabilization stage, biomolecules, including proteins, tannins, flavonoids, or polysaccharides, adsorb onto the nanoparticle surfaces, preventing aggregation and influencing morphology. Techniques like FTIR and XPS often confirm the presence of these organic compounds on the nanoparticle surfaces [52]. Numerous recent studies have demonstrated the biosynthesis of Cu/CuO-NPs using both plants and microorganisms [49,50,53,54,55,56]. Figure 1 shows the schematic representation of biological synthesis, characterization, and potential antimicrobial and anticancer applications of Cu/CuO-NPs.

### 2.1. Plant Derived Copper/Copper Oxide Nanoparticles

Plants are known as nature’s chemical factories due to their low cost and low care requirements [57]. Plants possess considerable potential for the uptake, sequestration, and detoxification of heavy metals, making them valuable biological agents for reducing environmental contamination [58]. Plant extract-based nanoparticle synthesis has several benefits over other synthesis techniques [59]. The rate of metal nanoparticle formation aided by plant extract is more durable, substantially faster, and highly monodisperse compared to other biological approaches [60]. Consequently, plant-derived extracts represent a highly promising and versatile biological resource for the sustainable synthesis of metal and metal oxide nanoparticles. Plant-assisted nanoparticle synthesis exhibits significantly faster reaction kinetics compared to other methods [61]. Fruit, leaves, stems, and roots are examples of plant parts that are commonly used for the environmentally friendly method of producing nanoparticles because of the superior phytochemicals they produce [62]. Due to the reasons stated above, copper oxide nanoparticles have been extensively produced using a range of plant-based extracts. To prepare a plant extract for nanoparticle production, the selected plant part (such as leaves, flowers, fruits, or seeds) is thoroughly washed, chopped into smaller pieces, and immersed in a solvent like ethanol or deionized water under controlled temperature for a set duration. Afterward, the solution containing the extract is filtered to remove plant residues, collected, and utilized as a natural reducing and stabilizing agent in nanoparticle formation [63]. In the green synthesis approach, metal salts such as copper nitrate, copper chloride, copper sulfate, etc., are combined with plant extracts, and the reaction typically proceeds for several hours at ambient temperature. These plant extracts are rich in bioactive compounds such as phenols, flavonoids, terpenoids, proteins, and tannins that function as both reducing and stabilizing agents, facilitating the conversion of metal ions into nanoparticles. Plant phytochemicals reduce copper ions, facilitating the formation of copper nanoparticles (Cu-NPs) and copper oxide nanoparticles (CuO-NPs) [64]. There is substantial research on plant-mediated synthesis of Cu/CuO-NPs and their potential antimicrobial and anticancer applications (Table 1).

**Figure 2:**
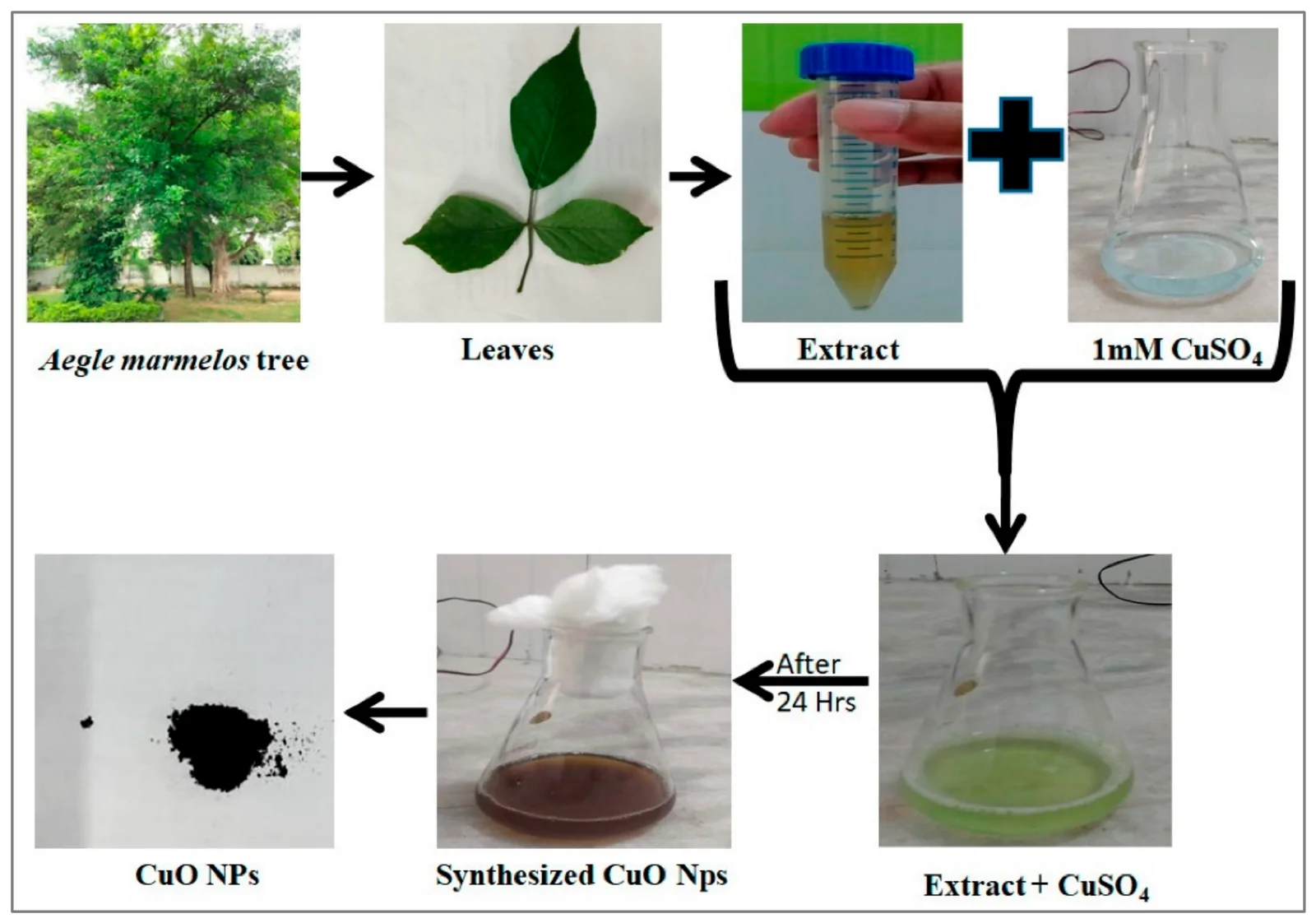
Biological synthesis of CuO-NPs using the leaves extract of the *Aegle marmelos* plant [65].

The leaf extract of the *Aegle marmelos* plant was used by Ali et al. [65] for the ecofriendly and facile synthesis of CuO-NPs (Figure 2). The leaf extract (10 mL) of *A. marmelos* was mixed with 90 mL of 1 mM copper (II) sulfate solution. The resulting mixture was stirred continuously for 24 h, during which a noticeable color change occurred. After this duration, the solution underwent centrifugation at speeds ranging from 10,000 to 12,000 rpm, leading to the formation of a solid pellet. This pellet was then dried and subjected to various characterization techniques. Finally, the well-characterized CuO-NPs were utilized for the treatment of different pathogenic bacteria and fungi [65].

Similarly, the leaf extract of *Passiflora edulis* was used by Yasin et al. [66] for the biological synthesis of CuO-NPs. A mixture of 60 mL leaf extract and 2 g copper nitrate trihydrate was stirred and heated at 80 °C until a green paste formed. The paste was filtered, washed 2–3 times with distilled water, and calcined in a silica crucible at 250 °C for 1 h in a muffle furnace. The resulting dark powder was obtained as CuO-NPs [66]. Several physicochemical factors including the plant species used, extract composition, salt and extract concentrations, the ratio of extract to salt, duration and temperature of incubation, as well as pH, play crucial roles in determining the yield, morphology, size, and stability of the synthesized Cu/CuO-NPs [49,67,68]. According to Moreno-Samaniego et al. [69], CuO-NPs of 30 to 300 nm in size with an oval shape were synthesized using the leaf extract of *Eucalyptus globulus*. On the other hand, Bala et al. [70], synthesized CuO-NPs of 20 to 36 nm with spherical shapes using leaf extract of *Oxalis corniculata*.

**Table 1:** Plant-derived copper/copper oxide nanoparticles and their potential antimicrobial and anticancer applications.

Plant	Used Part	Precursor	Shape, Size	Target Pathogens/Cancers	Reference
*Lonicera japonica*	Plant extract	Copper chloride	Spherical, 2–4 nm	*S. aureus*, *E. coli*, *Aspergillus niger*, and *C. albicans*	[46]
*Searsia lancea*	Fruit extract	Copper sulphate pentahydrate	Spherical, 54.4 nm (average)	HEK293 and HeLa cancer cell lines	[71]
*Ficus pumila*	Leaf extract	Copper sulphate pentahydrate	Cubic to slightly spherical, 100–150 nm	*S. aureus*, *P. aeruginosa*, and human glioblastoma cell line	[72]
*Mentha longifolia*	Leaf extract	Copper nitrate	Spherical, 50–90 nm	MCF-7 (breast) and AGS (gastric) cancer cell lines	[73]
*Annona muricata*	Plant extract	Copper nitrate	Spherical, 33.24 ± 6.49 nm	Breast cancer cell lines	[14]
*Cassia fistula*	Leaves	Copper sulphate pentahydrate	Spherical, 12–38 nm	*Fusarium oxysporum*	[74]
*Albizia saman*	Leaves	Copper sulphate pentahydrate	Spherical, 30–90 nm	*S. aureus*, *E. coli*, and *C. albicans*	[75]
Orange	Peel extract	Copper nitrate	Irregular, spherical, 50 nm (average)	Tobacco mosaic virus	[34]
*Cardaria draba*	Leaves	Copper nitrate	Spherical, 24–67 nm	*S. aureus*, *E. coli*, and breast cancer cells (MCF7)	[76]
*Acalypha Indica*	Leaf extract	Copper sulfate	Spherical	Human colon cancer cell lines (HCT-116)	[77]
*Aloe vera*	Leaves	Copper nitrate trihydrate	Sago-shaped, 15.8 nm (average crystalline size)	*S. typhi*, *Pseudomonas aeruginosa*, *Klebsiella pneumoniae*, and *Listeria monocytogenes.*	[49]
*Tectona grandis*, *Lantana camara*, *Azadirachta indica*, and *Senna spectabilis*	Leaves	Copper sulphate pentahydrate	Flake-like agglomerated cluster, less than 100 nm	*Corynespora cassiicola*	[78]
*Juglans regia*	Husk extract	Copper acetate	28 nm (average)	Herpes simplex virus types 1	[79]
*Rumex vesicarius*	Leaf extract	Copper acetate monohydrate	Spherical and irregular, 5–200 nm	Human cervical carcinoma (HeLa) cells	[80]
*Camellia sinensis*	Leaf extract	Copper nitrate	Spherical, 20–60 nm	Human colon and breast cancer cells	[81]
*Asparagus adscendens*	Root and leaf extract	Copper sulphate pentahydrate	Spherical, 10–60 nm	*Salmonella typhi*, *Bacillus subtilis*, *K. pneumonia*, *E. coli*, and *S. aureus*	[35]
*Citrus medica*	Fruit	Copper sulphate pentahydrate	Crystalline, 10–60 nm	*P. aeruginosa*, *K. pneumoniae*, *Propionibacterium acnes*, *Salmonella typhi*, *E. coli*, *Fusarium oxysporum* and *Fusarium graminearum.*	[82]
*Citrus sinensis*	Fruit	Copper sulphate pentahydrate	Roughly round in shape, 6.93–20.70 nm	*E. coli* and *S. aureus*	[83]
*Mentha spicata*	Leaves	Copper nitrate	Spherical, 10–47 nm	OECM-1 cancer cells	[84]
*Oxalis corniculata*	Leaves	Copper sulfate	Spherical, 20–36 nm	*E. coli* and *S. aureus*	[70]
*Bauhinia variegate* and *Saussurea lappa*	Flower	Copper sulfate	Spherical, 8.721 and 98.03 nm (average)	*E. coli*, *P. aeruginosa*, *K. pneumoniae*, *S. aureus*	[36]
*Hibiscus rosa-sinensis*	Flowers	Copper acetate	Spherical, 45–80 nm	*B. subtilis*, *K. pneumoniae*, *E. coli*, *S. aureus*	[85]
*Nilgirianthus ciliatus*	Leaves	Copper sulfate	Spherical, 20 nm (average)	Human breast cancer cell line (MCF-7) and lung cancer cell line (A549)	[86]
*Coriandrum sativum*	Leaves	Copper sulfate	Spherical, 13–14 nm	Oral carcinoma KB cells	[41]
Walnut shell	Powder	Copper nitrate hexahydrate	Rod-like nanoparticle, under 100 nm	Breast and colorectal cancer cell lines	[87]
*Zingiber officinalis* and *Curcuma longa*	Rhizome	Copper sulfate	Spherical, 20–100 nm	*S. aureus*	[88]
*Nigella sativa*	Seeds	Copper sulphate pentahydrate	98.23 nm	*P. aeruginosa*, *E. coli*	[68]
*Eucalyptus globulus*	Leaves	Copper sulphate pentahydrate	Oval, 30–300 nm	*E. coli*, *P. aeruginosa*, *K. pneumoniae*, *Salmonella enterica*, *Salmonella paratyphi*, *Edwarsiella tarda*, *Shigella flexneri*, *Aeromonas hydrophila*, *S. aureus*, *Proteus mirabilis*, and *Enterococcus faecalis*	[69]
*Zea mays*	Dry husk	Copper acetate monohydrate	Spherical and conical shaped, 10–90 nm	*E. coli*, *P. aeruginosa*, *S. aureus*, and *Bacillus licheniformis.*	[89]
*Nerium oleander*	Leaves	Copper chloride	Cylindrical shape, around 30 nm.	*E. coli* and *S. typhi* bacteria	[90]
*Punica granatum*	Peel extract	Copper acetate monohydrate	Spherical, 12.5 nm (average)	*E. coli*	[91]
*P. granatum*	Peel extract	Copper sulphate	Spherical, 15–20 nm	*P. aeruginosa*, *Micrococcus luteus*, *Salmonella enterica* and *Enterobactor aerogenes*	[92]
*Ocimum sanctum*	Leaves	Copper sulphate pentahydrate	Spherical, 73.50 nm (average)	*E. coli*, *P. aeruginosa*, *S. aureus*, *B. subtilis*	[93]
*Camella sinensis*	Leaves	Copper sulphate pentahydrate	Irregular, spherical, the average crystallite sizes were 5.9, 9.2, 11.5 and 15.5 nm	*E. coli*, *S. aureus*, *K. pneumoniae*, *Streptococcus mutans*	[94]
*Sida acuta*	Leaf	Copper sulfate	Nanorods, 50 nm (average)	*E. coli*, *S. aureus* and *Proteus vulgaris*	[95]
*Tridax procumbens*	Leaf extract	Copper sulfate	Irregular shape, 16 nm (average)	*Aedes aegypti* species	[96]
*Cissus quadrangularis* and *Piper betle*	Leaves	Copper sulfate	Spherical, 32.54 and 32.09 nm (average)	*Enterobacter* sp., *Flavobaterium psychrophilum*, *E. coli*, and *Klebsiella* sp.	[97]
*Ocimum tenuiflorum*	Plant	Coppe acetate monohydrate	Spherical, oval, and grain, nano-spheres (6–12 nm) and nano-rods (4–8/12–44 nm diameter/length)	*B. subtilis*, *E. coli*, *S. aureus*	[33]
*Ocimum americanum*	Leaves	Copper sulphate pentahydrate	Spherical, around 68 nm	*E. coli*, *A. hydrophila*, *Vibrio cholerae*, *K. pneumoniae*, *Salmonella* Typhimurium, and *P. aeruginosa*	[98]
*Syzygium aromaticum*	Bud extract	Cupric acetate	Spherical, around 20 nm (average)	*E. coli*, *Staphylococcus* spp., *Bacillus* spp., *Aspergillus flavus Pseudomonas* spp., *A. niger*, and *Penicillium* spp.	[99]
*Syzygium alternifolium*	Stem bark	Copper sulphate pentahydrate	Spherical, 5–13 nm	*B. subtilis*, *E. coli*, *S. aureus*, *K. pneumonia*, *Proteus vulgaris*, *Salmonella* Typhimurium, *P. aeruginosa*, *Alternaria solani*, *A. flavus*, *Penicillium chrysogenum*, *A. niger*, and *Trichoderma harzianum*	[40]
*Salvinia cucullata*	Leaves	Copper sulfate	Spherical, 18.23 nm (average)	*B. subtilis*, *P. aeruginosa*, *S. aureus*, *Enterococcus* spp., *K. pneumonia*, *S. aureus*, *E. coli*, *A. hydrophila*	[100]
*Rubia cordifolia*	Bark	Copper sulfate	Spherical, 50.72 nm (average)	*E. coli*, *S. aureus*, *P. aeruginosa*, and *Bacillus cereus*	[101]
*Morinda citrifolia*	Leaves	Copper sulphate pentahydrate	Spherical, 20–50 nm	*B. subtilis*, *S. aureus*, *E. coli*, *A. niger*, *A. flavus*, and *P. frequentans*	[102]
*Capsicum frutescens*	Leaves	Copper nitrate	Rectangular-rod 20–40 nm	*Bacillus anthracis*, *K. pneumoniae*, *L. monocytogenes*	[103]
*Psidium guajava*	Leaves	Copper sulfate	Oval and platelets, 40–150 nm	*E. coli*, *S. pneumoniae*, *P. aeruginosa*, *Staphylococcus epidermidis*	[104]
*Allium sativum*	Raw *Allium sativum*	Cupric nitrate	Spherical, oval-shaped, 20–50 nm	*Anopheles subpictus*	[105]
*Ficus racemosa*	Leaves	Copper sulphate	Spherical, 30–300 nm	*B. cereus*, *Clostridium perfringens*, *Campylobacter jejuni*, *B. subtilis*, *S. aureus*, *Vibrio cholerae*, *E. coli*, *Streptococcus pyogenes*, *Shigella dysenteriae*, *Helicobacter pylori*, *L. monocytogenes*, and *S. typhi*	[106]
*Madhuca longifolia*	Flowers and seeds	Cupric nitrate	Spherical and irregular, 30–120 nm	*S. aureus* and *B. subtilis*	[107]
*Gloriosa superba*	Leaves	Cupric nitrate	Spherical, 5–10 nm.	*Klebsiella aerogenes*, *S. aureus*, *E. coli*, *Pseudomonas desmolyticum*	[108]

### 2.2. Microbe Derived Copper/Copper Oxide Nanoparticles

Recently, both bacteria and fungi have been employed in the biosynthesis of various nanoparticles, including copper and copper oxide nanoparticles [109,110,111]. There is substantial research on microbe-mediated synthesis of Cu/CuO-NPs and their potential antimicrobial and anticancer applications (Table 2). Extracellular and intracellular techniques are commonly used for the easy and ecofriendly synthesis of Cu/CuO-NPs using microorganisms. Both microbial extract and culture supernatant contain various proteins, enzymes, peptides, polysaccharides, as well as other secondary metabolites that function as reducing, capping, and stabilizing agents throughout the nanoparticle synthesis process. These biomolecules aid in converting metal ions into nanoparticles and inhibit their aggregation, thereby significantly influencing the size, shape, and stability of the synthesized nanoparticles. According to Saitawadekar et al. [112], culture supernatant of *Aspergillus flavus* was utilized for the biological synthesis of bioactive CuO-NPs.

**Figure 3:**
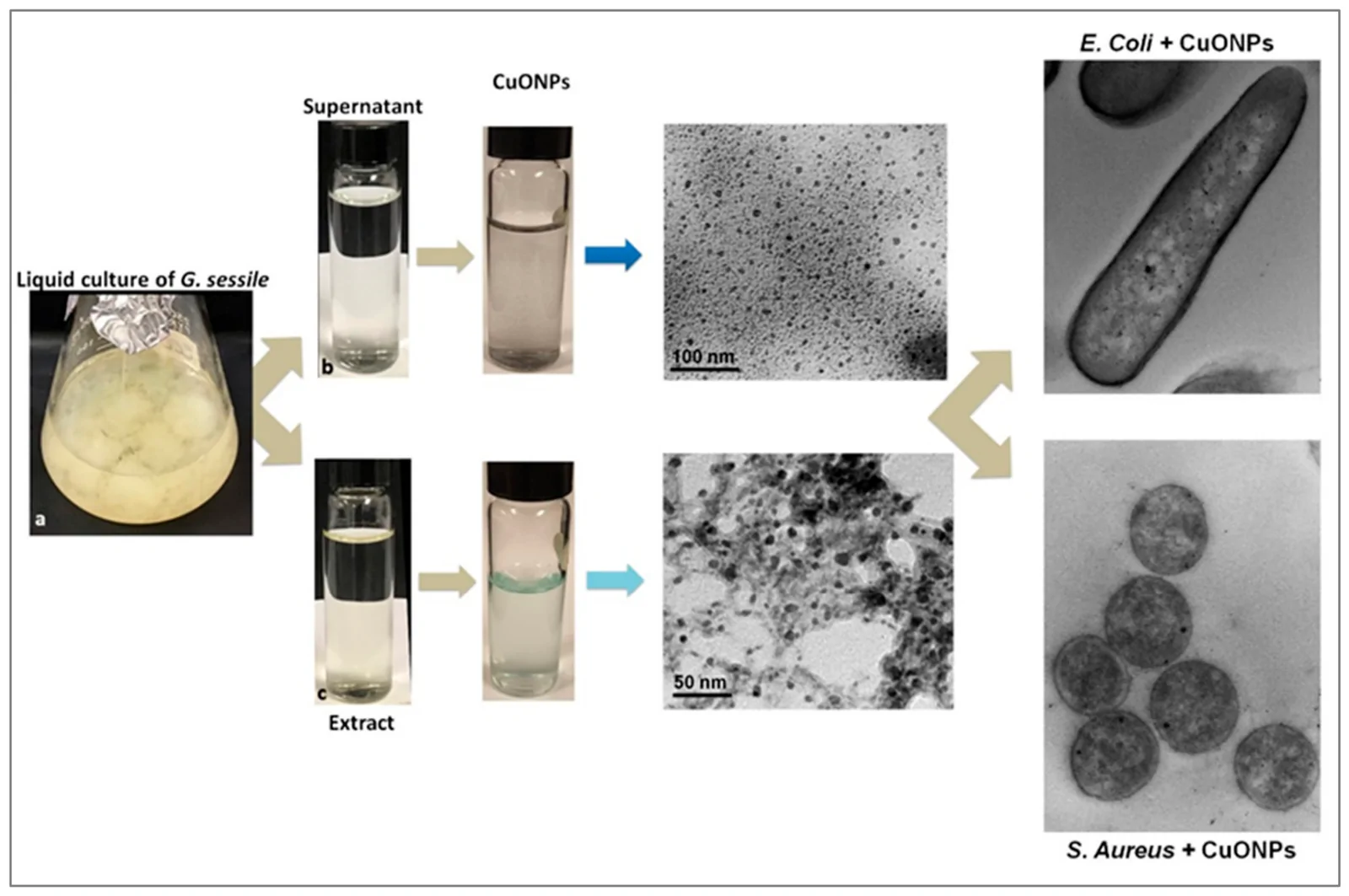
Biological synthesis of antibacterial active CuO-NPs using both culture supernatant and cell extract of the fungus *Ganoderma sessile* [115]. (**a**) Liquid culture of *G. sessile*; (**b**) supernatant; and (**c**) extract.

Kouhkan et al. [113] reported the extracellular biosynthesis of CuO-NPs using *Lactobacillus casei*, resulting in spherical nanoparticles ranging from 10 to 40 nm in diameter. Similarly, Nassar et al. [114] utilized the culture supernatant of *Aspergillus terreus* for CuO-NP synthesis and observed particles with spherical morphology, measuring between 15 and 55 nm. Noor et al. [109] also documented the bioreduction of CuSO_4_ to form CuO-NPs using the culture supernatant of *Aspergillus niger*. In another study, Flores-Rábago et al. [115] employed both the culture supernatant and cell extract of the fungus *Ganoderma sessile* for the green synthesis of CuO-NPs. Figure 3 illustrates the schematic overview of the biological synthesis of CuO-NPs using *Ganoderma sessile*, along with their characterization and antimicrobial activity against pathogenic bacteria such as *Escherichia coli* and *Staphylococcus aureus*. In this process, copper (II) sulfate pentahydrate (5 mM) was combined with either the fungal supernatant or extract in a 3:1 ratio. After adjusting the pH to 10 with 10 mM NaOH, a noticeable color shift from clear to light blue or blue-grey occurred. The mixture was then incubated at 60 °C for 24 h. Following incubation, the resulting pellets were isolated via centrifugation and freeze-dried to yield a fine nano powder. The resulting CuO-NPs were utilized for characterizations using different instruments including TEM as well as for the antimicrobial applications against pathogenic strains including *Escherichia coli*, and *Staphylococcus aureus.* TEM images display the synthesized CuO-NPs at varying magnifications. The 50 nm scale bar offers a closer view of structural details compared to the broader features observed at the 100 nm scale (Figure 3) [115].

**Table 2:** Microbe-derived Copper/copper oxide nanoparticles and their potential antimicrobial and anticancer applications.

Microbe	Synthesis Method	Precursor	Shape, Size	Target Pathogens/Cancers	Reference
*Ganoderma sessile*	Intracellular and extracellular	Copper sulfate pentahydrate	Quasi-spherical, 1–15 nm	*Escherichia* *coli*, *Pseudomonas aeruginosa*, *Staphylococcus aureus*	[115]
*Bacillus coagulans*	Extracellular	Copper sulfate	Spherical, 5–47 nm	MCF-7 and SKBR3 cancer cells	[116]
*Trichoderma* strains	Extracellular	Copper sulfate pentahydrate	Irregular shape, 1.3–160 nm	*Candida albicans*	[117]
*Trichoderma* *asperellum*	Extracellular	Copper nitrate	Spherical, 10–190 nm	Human lung cancer	[118]
*Stenotrophomonas* sp. BS95	Extracellular	Copper sulfate pentahydrate	Spherical, 35.24 ± 4.64 and 43.68 ± 2.31 nm (average)	*Bacillus subtilis*, *Pseudomonas putida*, *S. aureus*, and *E. coli*, human colon and gastric adenocarcinoma cells	[37]
*Streptomyces zaomyceticus* and *Streptomyces pseudogriseolus*	Intracellular	Copper sulfate pentahydrate	Spherical, 78 to 80.0 nm (average)	*Bacillus diminuta*, *S. aureus*, *Pseudomonas aeruginosa*, *Bacillus subtilis*, *E. coli*, *Aspergillus brasiliensis*, and *C. albicans*	[119]
*Actinomycetes* spp.	Extracellular	Copper sulfate pentahydrate	Spherical, 61.7 nm	*S. aureus*, *Aeromonas hydrophila*, *Bacillus cereus*, *Vibrio anguillarum*, *Proteus mirabilis*, *Aeromonas caviae*, *Edwardsiella tarda*	[120]
*Aspergillus niger*	Extracellular	Copper sulfate	Spherical, 5–100 nm	*E. coli*, *Klebsiella pneumonia*, *S. aureus*	[109]
*Streptomyces* sp. MHM38	Extracellular	Copper sulfate	Spherical, 1.72–13.49 nm	*Enterococcus faecalis*, *Salmonella* Typhimurium, *E. coli*, *P. aeruginosa*, *Fusarium solani*, *Rhizoctonia solani*, *A. niger* and *C. albicans*	[121]
*Halomonas elongata* IBRC-M 10214	Extracellular	Copper sulfate	Rectangular, 57–79 nm	*E. coli*, and *S. aureus*	[122]
*Aspergillus terreus*	Extracellular	Copper sulfate pentahydrate	Spherical, 15–55 nm	*B. subtilis*, *E. coli*, *S. aureus*, *P. aeruginosa*, and various *Candida* species (*C. glabrata*, *C. parapsilosis*, *C. albicans* and *C. tropicalis*), Human breast cancer (MCF7), and prostate cancer (PC3) cells,	[114]
*Shewanella loihica* PV-4	Extracellular	Copper chloride	Spherical, 6–20 nm	*E. coli*	[123]
*B. cereus*	Extracellular	Copper sulfate	Spherical, around 50 nm (average)	*B. subtilis*, *S. aureus*, *E. coli*, and *P. aeruginosa*	[124]
*Brevibacillus brevis*	Extracellular	Copper acetate monohydrate	Spherical, 2–28 nm	*Fusarium oxysporum*, *A. niger*, *Alternaria alternata*, and various clinical *Candida* spp.	[125]
*Gluconacetobacter hansenii*	Bacterial cellulose	Copper nitrate	Irregular shape, 25–35 nm	*S. aureus*, *Salmonella enterica*, and *C. albicans*	[126]
*Lactobacillus casei*	Extracellular	Copper sulfate	Spherical, 40–110 nm	*S. aureus* and *P. aeruginosa*	[113]
*Morganella morganii*	Extracellular	Copper sulfate	Spherical, 13.5 ± 0.6 nm (average)	*B. Subtilis*, *E. coli*, *Metarhizium anisopliae*, *A. niger*, and *Verticillium* sp.	[127]
*Penicillium chrysogenum*	Extracellular	Copper sulfate pentahydrate	Spherical, 9.70 nm (average)	*F. oxysporum*, *A. niger*, *Ralstonia solanacearum*, *Alternaria solani* and *Erwinia amylovora*	[128]
*Actinomycetes*	Extracellular	Copper sulfate pentahydrate	Spherical, 10 to 30 nm	*E. coil*, *P. miarbilis*	[129]
*Microbacterium liquefaciens*	Extracellular	Copper sulfate	Hexagonal, 31.29 nm (average)	*E. coli* and *S. aureus*	[130]
*Streptomyces cyaneus*	Melanin pigment	Copper sulfate pentahydrate	Spherical, 22.2 nm to 34.4 nm	*B. subtilis*, *L. monocytogenes*, *E. coli*, *S.* Typhimurium, *S. aureus*, *A. niger*, *F. oxysporum*, *Penicillium citrinum*, and *Alternari chlamydosporum*	[131]
*Trichoderma harzianum*	Extracellular	Copper sulfate pentahydrate	Elongated fibers in shape, 38 and 77 nm in width and 135–320 nm in length	*Pleomorphomonas oryzae*, *A. alternata*, and *Sclerotinia* *sclerotiorum*	[132]
*Bacillus subtilis*, *M. morganii*	Extracellular	Copper sulfate	Spherical, less than 10 nm	*Streptococcus pneumoniae*	[133]
*Aspergillus fumigatus*	Intracellular	Copper nitrate	Spherical, up to 48 nm	*S. aureus*, *K. pnemonia*	[134]

## 3. Characterization of Cu and CuO Nanoparticles

The accurate characterization of copper (Cu) and copper oxide (CuO) nanoparticles is essential for understanding their structural, morphological, and physicochemical properties, which influence their functional performance in biomedical, environmental, and catalytic applications. As Cu and CuO nanoparticles differ in oxidation states (Cu^0^, Cu^+^, and Cu^2+^), crystal structures (face-centered cubic vs. monoclinic), and electronic properties, a combination of advanced analytical techniques is required for comprehensive evaluation. Ali et al. [65] explored the utilization of leaf extract of *A. marmelos* in the eco-friendly synthesis of CuO-NPs. The synthesized CuO-NPs were extensively characterized using different techniques, including UV-Vis spectroscopy, SEM, TEM, EDX, and XRD [65]. SEM confirmed that the CuO-NPs were well dispersed without aggregation, while EDX verified elemental copper, TEM showed an average particle size of 32 nm, UV–Vis exhibited a characteristic absorption peak at 330 nm, and XRD confirmed their crystalline nature [65]. UV-vis, SEM, FTIR, and XRD techniques were used by Yasin et al. [66] to examine the biologically produced CuO-NPs using the leaf extract of *P. edulis*. UV–visible spectroscopy showed an absorbance peak at 249.9 nm, confirming the presence of CuO-NPs. The scanning electron microscope (SEM) reveals the surface structure of the synthesized CuO-NPs. The SEM images indicate a predominantly spherical shape of synthesized CuO-NPs with slight agglomeration, and the average particle size ranges from 60 to 65 nm. XRD analysis confirms the crystalline nature of biosynthesized CuO-NPs. FTIR analysis revealed that the synthesized nanoparticles are not composed of pure CuO alone; instead, they possess an organic coating originating from compounds present in the *P. edulis* extract [66].

**Figure 4:**
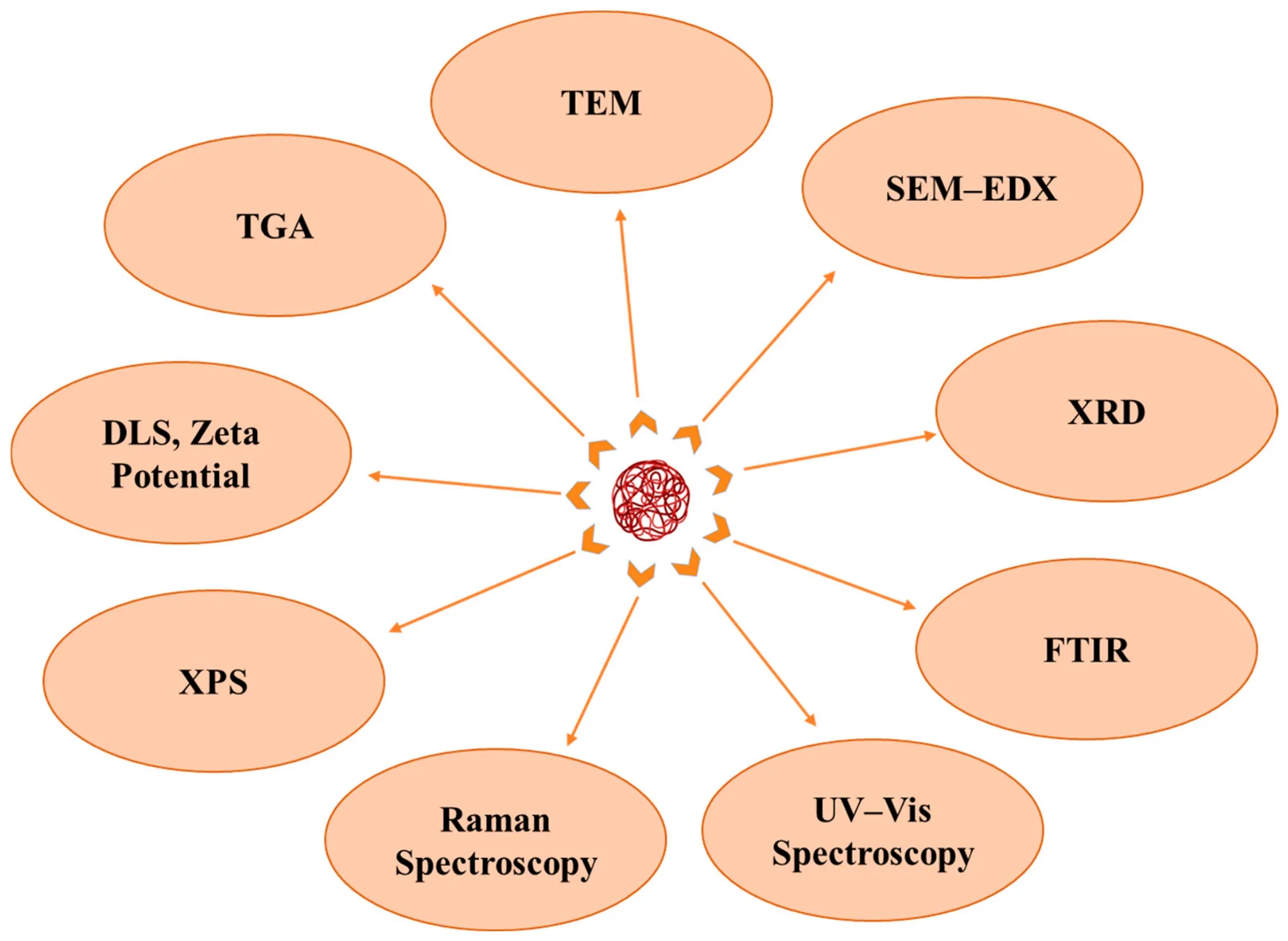
Schematic representation of the characterization techniques employed for Cu and CuO nanoparticles, including Transmission Electron Microscopy (TEM), Scanning Electron Microscopy with Energy Dispersive X-ray Analysis (SEM–EDX), X-ray Diffraction (XRD), Fourier Transform Infrared Spectroscopy (FTIR), Ultraviolet–Visible Spectroscopy (UV–Vis), Raman Spectroscopy, X-ray Photoelectron Spectroscopy (XPS), Dynamic Light Scattering (DLS), Zeta Potential and Thermogravimetric Analysis (TGA). These methods collectively provide insights into nanoparticle morphology, crystallinity, functional groups, surface charge, stability, elemental composition, and thermal behavior.

According to Flores-Rábago et al. [115], *G. sessile* fungus-mediated green synthesized CuO-NPs were characterized by UV-Vis spectroscopy, TEM, FTIR, XRD, DLS, and zeta potential analysis. UV–Vis spectroscopy showed an absorption peak at 290.73 nm, while DLS indicated a polydisperse distribution (PDI = 0.619), TEM revealed quasi-spherical nanoparticles with sizes of 1–15 nm, and zeta potential analysis showed good colloidal stability (−28.7 mV). XRD analysis suggested that the nanoparticles possessed an amorphous structure, while FTIR confirmed the presence of functional groups involved in nanoparticle formation [115]. Liu et al. [34] used SEM, TEM, XPS, XRD, and FTIR to characterize the biosynthesized CuO-NPs using the orange peel extract. SEM and TEM analyses revealed irregularly spherical CuO-NPs with an average size of approximately 50 nm, while XRD confirmed their crystalline structure. XPS and FTIR analyses characterized the elemental composition and surface chemistry of the synthesized CuO-NPs. FTIR characterization identified alcohol- and phenol-related functional groups associated with the synthesized nanoparticles. XPS analysis confirmed the surface composition of the CuO-NPs, revealing prominent C, O, and Cu signals indicative of their elemental makeup [34]. Garcia-Marin et al. [117] used UV-Vis spectroscopy, TEM, DLS, zeta potential analysis, and FTIR to characterize biosynthesized CuO-NPs using *Trichoderma* strains. TEM analysis indicated that the synthesized CuO-NPs were polydisperse. Measurements of 1000 individual nanoparticles revealed two distinct size distributions. The majority of the particles were quasi-spherical, with sizes ranging from 1.3 to 30 nm, and an average diameter of 5.8 ± 4.8 nm. A smaller fraction (8.4%) exhibited varied shapes and were significantly larger, measuring between 30 and 160 nm, with an average size of 54.1 ± 23.5 nm. UV–Vis spectroscopy showed an absorbance peak at 290 nm, confirming the presence of CuO-NPs. FTIR spectroscopy identified several functional groups responsible for the reduction and stabilization of the nanoparticles [117]. Nanoparticles of different sizes may form even under identical experimental conditions, as the synthesis process is highly sensitive to minor variations. Small fluctuations in temperature, pH, precursor concentration, or stirring speed can alter nucleation and growth dynamics, resulting in size differences. To solve this problem, it is essential to rigorously regulate and monitor experimental variables by using high-grade reagents, maintaining accurate temperature and pH control, optimizing precursor-to-stabilizer ratios, allowing sufficient reaction time for complete growth, and applying advanced methods such as microreactors to achieve tighter control over reaction parameters [135,136,137]. Figure 4 illustrates the different techniques commonly used for the characterization of biosynthesized Cu/CuO-NPs.

### 3.1. X-Ray Diffraction (XRD)

XRD is a primary tool for identifying the crystalline structure and phase purity of nanoparticles. It is based on Bragg’s Law, which relates the wavelength of incident X-rays to the diffraction angle and interatomic spacing in a crystalline sample [138]. For Cu and CuO nanoparticles, XRD allows differentiation between metallic copper (Cu^0^), cuprous oxide (Cu_2_O), and cupric oxide (CuO) based on their characteristic diffraction peaks. For example, CuO typically exhibits a monoclinic structure with diffraction peaks at 2θ values around 32.5°, 35.5°, and 38.7°, while Cu^0^ exhibits peaks at ~43.3°, 50.4°, and 74.1° corresponding to the (111), (200), and (220) planes of the face-centered cubic lattice [139].

### 3.2. Transmission Electron Microscopy (TEM)

TEM provides direct visualization of nanoparticles at the nanometer and even atomic scale. It reveals key features such as size, shape, lattice fringes, and aggregation state. In the case of Cu and CuO nanoparticles, TEM has been widely used to identify spherical or quasi-spherical morphologies and sizes ranging from 10 to 100 nm, depending on the synthesis route. The lattice spacing observed in high-resolution TEM can also be used to confirm crystallographic planes characteristic of Cu or CuO [140].

### 3.3. Scanning Electron Microscopy (SEM) and Energy Dispersive X-Ray Spectroscopy (EDX)

Scanning Electron Microscopy (SEM) is widely used to evaluate the surface morphology and particle topography of copper-based nanoparticles. SEM images typically reveal that Cu and CuO nanoparticles possess spherical, quasi-spherical, or hexagonal morphologies, often with rough surfaces and varying degrees of aggregation depending on the synthesis method [141,142]. These morphological characteristics are crucial for understanding nanoparticle behavior in applications such as antimicrobial coatings or catalysis. Coupled with EDX, SEM becomes a powerful tool for elemental analysis. EDX spectra commonly confirms the presence of copper (Cu) and oxygen (O), supporting the successful formation of CuO or Cu nanoparticles. In addition to confirming elemental composition, EDX can detect impurities or validate whether the synthesized material is Cu-rich or O-rich critical for ensuring the purity and stoichiometry of the nanoparticles [142].

### 3.4. Fourier Transform Infrared Spectroscopy (FTIR)

FTIR spectroscopy is a key tool for identifying functional groups on the surface of CuO nanoparticles and confirming metal–oxygen bonding. In both chemical and green synthesis routes, Cu–O stretching vibrations consistently appear in the range of 500–600 cm^−1^, confirming the formation of copper oxide. For example, green-synthesized CuO-NPs using extract from *C. persicus* leaves exhibited characteristic Cu–O bands at 522 cm^−1^ and 590 cm^−1^, alongside additional peaks indicative of organic groups: 3425 cm^−1^ (–OH stretching), 1629 cm^−1^ (C=O), 1470 cm^−1^ (aromatic C=C), and 1073 cm^−1^ (C–O) [143]. Similarly, CuO-NPs capped with *Parthenium hysterophorus* extract showed FTIR peaks at 522 cm^−1^ and 590 cm^−1^, confirming Cu–O bonds; other observed bands include 3284 cm^−1^ (O–H), 1600 cm^−1^ (C=O of amide), 1368 cm^−1^ (C–N of amine), and 1084 cm^−1^ (flavonoid C–O) [144].

### 3.5. Ultraviolet–Visible (UV–Vis) Spectroscopy

UV–Vis spectroscopy is a widely used technique to confirm the formation of copper-based nanoparticles and to assess their optical properties. This method helps distinguish between metallic copper (Cu) and copper oxide (CuO) nanoparticles based on their unique absorption profiles. Metallic copper (Cu) nanoparticles typically exhibit a distinct surface plasmon resonance (SPR) peak in the visible region, generally between 560–590 nm, due to collective oscillations of conduction electrons on the nanoparticle surface when excited by light, while CuO-NPs have a different SPR band, usually between 250–400 nm [145,146,147]. This SPR band is a characteristic indicator of metallic nanoparticle formation and is strongly influenced by size, shape, and surrounding medium.

### 3.6. Dynamic Light Scattering (DLS) and Zeta Potential

The hydrodynamic diameter measures, reflecting the effective size of nanoparticles and any associated solvation or capping layer. It is invaluable for assessing aggregation in colloidal solutions, particularly in aqueous and biological environments. Changes in the hydrodynamic size indicate clustering or stability in suspension [148]. The hydrodynamic size, colloidal stability, and aggregation behavior of biosynthesized Cu/CuO-NPs are strongly influenced by the synthesis conditions and the nature of the biological reducing and capping agents. Parameters such as pH, temperature, precursor concentration, reaction time, and the concentration of plant extracts or microbial cultures can significantly affect nanoparticle nucleation, growth, and surface functionalization. Biomolecules including proteins, polysaccharides, polyphenols, flavonoids, amino acids, and other secondary metabolites adsorb onto the nanoparticle surface, forming a protective capping layer that increases the hydrodynamic diameter measured by DLS while simultaneously enhancing colloidal stability [149,150].

Zeta Potential: Indicates the electrostatic potential at the nanoparticle’s slipping plane, which is related to colloidal stability. Empirically:

- |ζ| > 30 mV (either positive or negative): Stable colloid—sufficient electrostatic repulsion to prevent aggregation.
- |ζ| 10–30 mV: Partial or moderate stability—weak repulsion may allow aggregation.
- |ζ| < 10 mV: Unstable, prone to flocculation/coagulation [151]

This threshold is widely accepted in nanoparticle research: zeta potentials exceeding ±30 mV are considered indicative of stable dispersions, making them particularly suitable for biomedical and environmental applications where aggregation must be minimized [148,152].

### 3.7. X-Ray Photoelectron Spectroscopy (XPS)

XPS is a highly surface-sensitive analytical technique used to determine the elemental composition and oxidation states of nanoparticles. It is especially valuable for distinguishing between different copper oxidation states in Cu/CuO nanoparticles based on their Cu 2p core-level spectra. Metallic copper (Cu^0^) typically shows a Cu 2p_3_/_2_ binding energy near 932.5 eV and does not exhibit satellite features. Cuprous oxide (Cu^+^, as in Cu_2_O) also presents a main peak around 932.5 eV, but with slightly different peak shapes and no significant satellite peaks. In contrast, cupric oxide (Cu^2+^, as in CuO) displays a Cu 2p_3_/_2_ peak at approximately 933.6 eV, accompanied by intense satellite peaks in the 940–945 eV region, which are characteristic of the Cu^2+^ state and result from shake-up transitions associated with the 3d^9^ electron configuration [153,154]. The presence of these satellite peaks is a definitive indicator of Cu^2+^ species and helps differentiate CuO from metallic copper or Cu_2_O. Therefore, XPS not only confirms the formation of copper oxide but also offers information regarding surface oxidation and the chemical environment of Cu/CuO nanoparticles [155].

### 3.8. Raman Spectroscopy

Raman spectroscopy provides valuable structural information on CuO nanoparticles by probing vibrational modes of the monoclinic CuO lattice. According to group theory, CuO (C_2_h symmetry) has three Raman-active modes: one Ag and two Bg modes. Characteristic peaks appear near 280–283 cm^−1^ (Ag), 330 cm^−1^ (Bg^1^), and 610–620 cm^−1^ (Bg^2^), which are consistently observed in CuO nanoparticle spectra [156]. These peaks serve as definitive markers for the presence of monoclinic CuO. Notably, Goraya & Singh (2016) reported Raman modes at ~283, 330, and 619 cm^−1^ for hydrothermally synthesized CuO nanocrystals [156]. Chakraborty et al. observed similar peaks (~280, 330, 620 cm^−1^) in CuO nanoparticles synthesized via a hydrothermal method [157]. Raman spectroscopy is especially useful for confirming structural integrity in hybrid or doped CuO systems.

### 3.9. Thermogravimetric Analysis (TGA)

TGA is a widely used technique for evaluating the thermal stability, composition, and surface chemistry of nanoparticles by measuring changes in mass as a function of temperature. This method is particularly useful for characterizing copper-based nanoparticles synthesized via green or chemical routes. In green synthesis approaches, CuO nanoparticles are often capped with plant-derived organic molecules, which are thermally unstable. TGA typically reveals a multi-step weight loss profile. The first weight loss stage, observed below 200 °C, corresponds to the evaporation of adsorbed moisture or volatile surface solvents. The second major weight loss, typically between 200–600 °C, is attributed to the thermal degradation of organic capping agents, such as polyphenols, flavonoids, or proteins present in plant extracts. This weight reduction serves as an indicator of the amount of biomolecules associated with the nanoparticle surface, confirming successful surface modification [158,159]. TGA is also effective in distinguishing between different copper oxidation states based on their thermal behavior. Metallic copper (Cu^0^) or cuprous oxide (Cu_2_O) may undergo oxidation or transformation upon heating, resulting in distinct mass changes. In contrast, CuO nanoparticles typically show minimal weight loss above 600 °C, reflecting their excellent thermal stability and phase purity [160]. Furthermore, the quantification of organic content based on total mass loss—often ranging between 10% and 45% in green-synthesized samples—provides insight into the degree of surface functionalization, which is crucial for applications in catalysis, antimicrobial and anticancer activities, and environmental remediation.

## 4. Biomedical Applications of Copper/Copper Oxide Nanoparticles

Cu/CuO NPs have attracted significant attention in biomedicine due to their antimicrobial, antioxidant, anticancer, and wound-healing properties. These nanoparticles exhibit strong activity against a wide range of bacteria, fungi, and viruses through the generation of reactive oxygen species and membrane disruption. Cu/CuO NPs are also explored for drug delivery, bioimaging, and cancer therapy because of their unique physicochemical characteristics and biocompatibility [14,27,39,161,162]. This review provides a critical overview of the antibacterial, antifungal, antiviral, and anticancer properties of green synthesized Cu/CuONPs. Special attention is given to the mechanistic pathways responsible for their biological activities.

### 4.1. Antibacterial Activity and Mechanisms of Copper/Copper Oxide Nanoparticles

**Figure 5:**
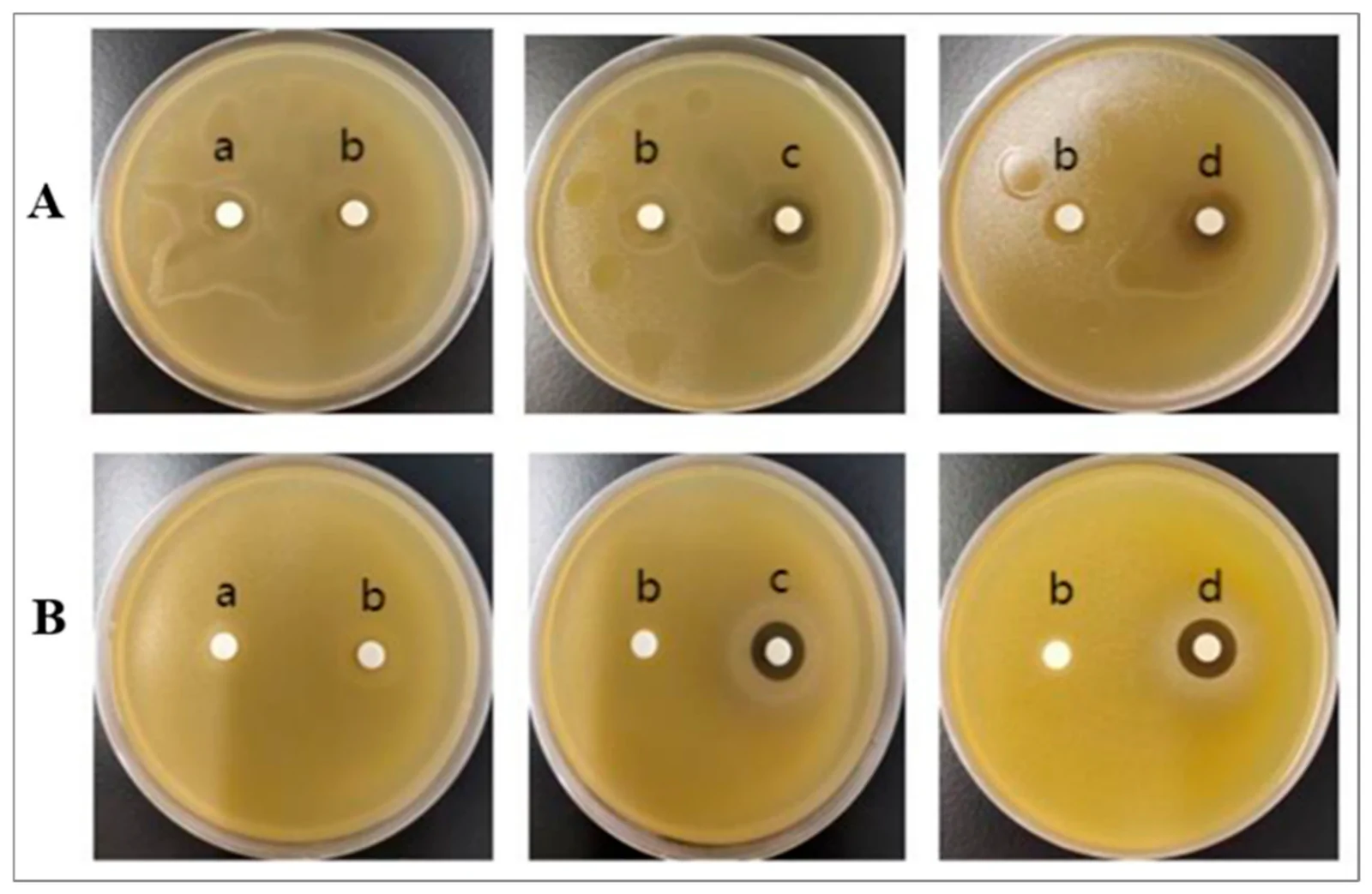
Zone of inhibition of *Lonicera japonica* Thunb extract mediated synthesized CuNPs on (**A**) *Escherichia coli*, and (**B**) *Staphylococcus aureus* plates. For all photographs, a represents the blank control group in PBS, b represents the sample of *L. japonica* Thunb extract, c represents 10 μg/mL, and d represents 20 μg/mL of the biosynthesized CuNPs [46].

Cu/CuO-NPs demonstrate strong antibacterial effects against both Gram-positive and Gram-negative bacterial strains. Many recent studies highlight the antibacterial efficacy of biosynthesized Cu/CuO-NPs against various bacteria (Table 1 and Table 2). These nanoparticles, synthesized using eco-friendly methods, demonstrate potent antimicrobial activity through various mechanisms, including the generation of reactive oxygen species, disruption of cell membranes, and interference with bacterial proteins and DNA [38,39,163]. Flores-Rábago et al. [115] reported fungus-mediated intracellular and extracellular synthesis of CuO-NPs and their antibacterial activity against pathogenic strains of *E. coli*, *Staphylococcus aureus*, and *Pseudomonas aeruginosa*. The synthesized CuO-NPs showed significant antibacterial activity against these pathogens. The minimum inhibitory concentration (MIC) values of extracellular and intracellular synthesized CuO-NPs against *E. coli* were 15.9 and 16.5 μg/mL, respectively. The MIC values of extracellular and intracellular synthesized CuO-NPs against *P. aeruginosa* were 13.7 and 16.5 μg/mL, respectively. The minimum bactericidal concentration (MBC) represents the lowest antimicrobial concentration capable of completely eliminating a bacterial population, while the minimum inhibitory concentration (MIC) is the lowest concentration that effectively prevents bacterial growth. Determining MIC and MBC values is essential for selecting appropriate antibiotics, improving patient recovery, assessing drug safety, minimizing resistance development, and supporting the creation of new antimicrobial agents. Another study focused on the biological synthesis of CuO-NPs using aqueous extract of *Lonicera japonica* Thunb and the antibacterial applications against pathogenic strains of Gram-positive *S. aureus* and Gram-negative *E. coli* [46]. The synthesized CuO-NPs showed a strong zone of inhibition (ZOI) against tested pathogens (Figure 5).

Priya et al. [102] demonstrated the biological synthesis of CuO-NPs using the leaf extract of *Morinda citrifolia* and evaluated their antimicrobial activity against *Bacillus subtilis*, *S. aureus*, *E. coli*, *A. niger*, *A. flavus*, and *Penicillium frequentans*. The flower and seed extract of *Madhuca longifolia* were employed in the green synthesis of CuO nanoparticles, which were subsequently evaluated for their antimicrobial potential against pathogenic *S. aureus* and *B. subtilis*. The results demonstrated that the synthesized CuO-NPs exhibited significant inhibitory effects on the tested microbial strains [107]. According to Manimehala et al. [94], *Camellia sinensis* leaf-mediated synthesized copper oxide nanoparticles showed strong antimicrobial activity against *P. aeruginosa*, and *C. albicans*. They control the pathogens by disrupting the cell membrane and generating ROS. Similarly, *Rubia cordifolia* bark-extract-mediated CuO-NPs demonstrated significant antimicrobial activity against *E. coli*, *S. aureus*, *P. aeruginosa*, and *Bacillus cereus* [101].

Mechanistically, CuO-NPs adhere to bacterial surfaces, destabilizing lipid bilayers and protein networks—a primary line of attack that leads to cytoplasmic leakage and cell lysis [164]. This contact-dependent disruption is rapidly followed by oxidative stress: Cu^2+^ ions leach from the nanoparticle surface and undergo redox cycling, generating reactive oxygen species (ROS) such as superoxide and hydroxyl radicals that damage lipids, proteins, and nucleic acids [39,165]. Studies have demonstrated that CuO-NPs produce ROS more efficiently than bulk copper compounds, due to their high surface-area-to-volume ratio and unique crystal facets [166,167]. In the context of enzyme inactivation, cuprous oxide (Cu_2_O) has been shown to bind and inactivate iron–sulfur cluster enzymes such as fumarase A, arresting bacterial metabolic functions [168]. Meanwhile, CuO-specific ROS production contributes additively, arresting bacterial growth at multiple metabolic junctures. This dual pathway—Cu_2_O-mediated enzyme complexation and CuO-driven ROS generation—reinforces the strong antibacterial efficacy of Cu-based nano systems. Empirical results from green-synthesized CuO-NPs illustrate this potency. For example, biogenic CuO-NPs produced using neem and jojoba extracts exhibited MICs of 62.5–125 µg/mL against MRSA, *Klebsiella pneumoniae*, *P. aeruginosa*, *Acinetobacter*, and *E. coli*, achieving bactericidal outcomes (MBC/MIC = 1) [164]. The same formulations reduced biofilm coverage by 62–95%, underscoring their anti-biofilm potential. Microscopy (TEM) imagery confirmed severe ultrastructural damage to cell walls and membranes, with nanoparticle-induced blebbing and intracellular content leakage visible after one hour of exposure [169]. The ability of CuO-NPs to mitigate biofilms is particularly significant. Biofilms confer substantial antibiotic tolerance, yet CuO-NPs were shown to inhibit biofilm formation in pathogens like *E. coli*, *S. aureus*, and *P. aeruginosa*, with over 70% reduction at sub-inhibitory concentrations. In plant pathogen studies, CuO-NPs eradicated *Ralstonia solanacearum* at 250 µg/mL and inhibited motility and ATP generation in these cells [165] demonstrating broad-spectrum applicability. Importantly, the physicochemical properties of CuO-NPs—size, morphology and surface charge—play critical roles in their antibacterial performance [39]. A seminal study by Azam et al. investigated the antibacterial activity of CuO nanoparticles of varying sizes. They synthesized CuO-NPs via a gel combustion method, annealing them at different temperatures to produce nanoparticles as small as ~20 nm. Their work demonstrated a clear size dependence: the smallest nanoparticles exhibited significantly higher antibacterial potency against both Gram-positive and Gram-negative bacteria compared to larger particles. The correlation was attributed to increased Cu^2+^ release and enhanced ROS generation due to the larger surface-area-to-volume ratio of smaller particles [170]. Despite the strong antibacterial potency, it is vital to address cytotoxicity and environmental safety. In vitro studies indicate that carefully optimized CuO-NPs (≤250 µg/mL) can achieve antimicrobial effects without significant cytotoxicity to mammalian cells. Composite formulations—e.g., CuO in chitosan or cellulose—have further enhanced biocompatibility and stability [39].

According to Flores-Rábago et al. [115], *G. sessile* mediated biosynthesized CuO-NPs effectively control the growth of pathogenic bacteria *E. coli*, and *S. aureus*. To examine the effects of CuO-NPs on bacterial cells, *E. coli* and *S. aureus* were treated at their respective MICs and subsequently examined by TEM. Nanoparticles were found distributed within the cell, with occasional small clusters observed. Flores-Rábago et al. [115] concluded that the antibacterial action of Cu/CuO nanoparticles involves multiple mechanisms, including membrane disruption, reactive oxygen species (ROS) production, ribosomal destabilization, mitochondrial impairment, lipid peroxidation, protein oxidation, and DNA damage.

### 4.2. Antifungal Activity and Mechanisms of Copper/Copper Oxide Nanoparticles

Cu/CuO-NPs are increasingly recognized for their potent antifungal properties, extending their antimicrobial utility beyond bacteria. Unlike conventional fungicides, Cu/CuO-NPs leverage unique nanoscale mechanisms—enhanced penetration, oxidative stress induction, and interference with fungal metabolism—offering effective control against a spectrum of fungal species, including drug-resistant strains and plant pathogens. The antifungal mechanism commences with nanoparticle-fungal interactions at the cell wall and membrane. CuO-NPs can breach chitin–β-glucan matrices, impairing membrane integrity and enabling intracellular ROS production. Similarly to bacteria, Cu_2_O facilitates enzyme binding, while CuO promotes ROS-mediated oxidative stress. In fungal cells, oxidative stress disrupts cell cycle regulation, mitochondrial function, and DNA integrity, leading to programmed cell death. Among pathogenic fungi studied, *Candida albicans*, *A. niger*, *Fusarium oxysporum*, *Penicillium chrysogenum*, and Tobacco mosaic virus (plant pathogen) are frequently inhibited by Cu-based NPs. Biosynthesized antimicrobial studies with CuO-NPs derived from citrus peel showed MICs of ~35 µg/mL against *C. albicans*, with ROS-induced intracellular damage confirmed through fluorescence microscopy and negligible mammalian cytotoxicity [169]. In agriculture, orange-peel-synthesized CuO-NPs at 100 mg/L caused fragmentation of TMV particles within two hours and reduced disease in tobacco plants [34] underscoring their potential for sustainable plant disease management.

**Figure 6:**
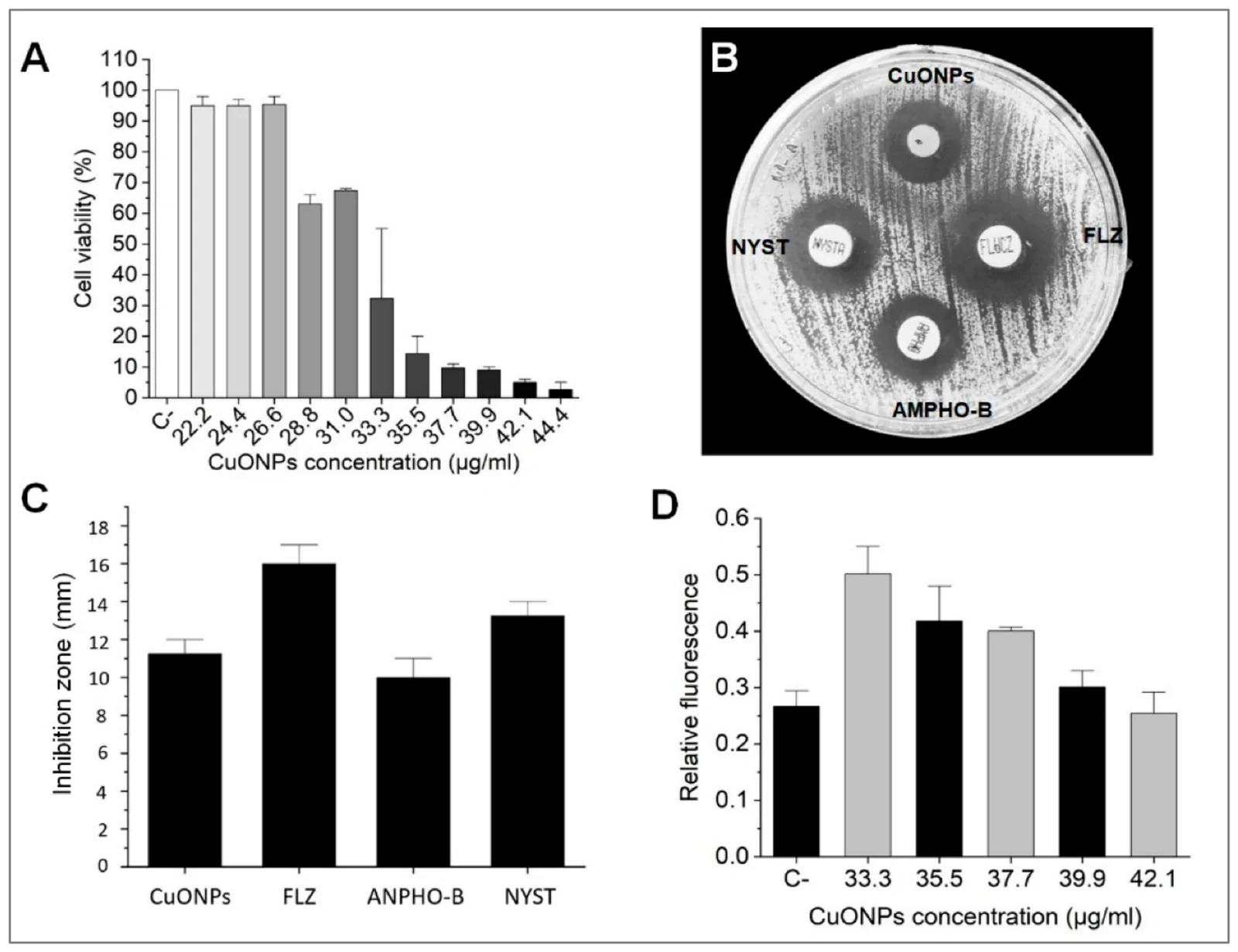
(**A**) Cell viability of *Candida albicans* following 48 h of incubation with varying concentrations of CuO-NPs for determining the MIC; (**B**) Comparison of *C. albicans* inhibition by CuO-NPs and standard antifungal agents; (**C**) Bar chart showing the diameters of inhibition zones from the antifungal susceptibility test after 24 h of incubation; (**D**) Reactive oxygen species (ROS) generation by *C. albicans* after 24 h of exposure to CuO-NPs. C- = negative control; FLZ = Fluconazole; ANPHO-B = Amphotericin B; NYST = Nystatin [117].

Garcia-Marin et al. [117] (2022) reported the biological synthesis of CuO-NPs using fungal extract and evaluated their antifungal efficacies against *C. albicans* (ATCC SC5314). The biosynthesized CuO-NPs effectively control the growth of pathogenic *C. albicans*. The MIC of synthesized CuO-NPs was 35.5 µg/mL against *C. albicans*. CuO-NPs, at a concentration of 35.5 µg/mL, exhibited comparable zones of inhibition to those produced by standard antifungal medications. Figure 6A–D shows the cell viability, inhibition zones, and ROS production by *C. albicans* after 24 h exposure to biosynthesized CuO-NPs [117]. Yu et al. [46] demonstrated the green synthesis of Cu-NPs using aqueous extract of *L. japonica* and evaluated their antifungal activity against *A. niger* and *C. albicans*. The synthesized Cu-NPs exhibited strong efficacy against the tested pathogens. According to Rajesh et al. [99], *Syzygium aromaticum* bud extract mediated synthesized CuO-NPs showed strong antifungal activity against *A. flavus*, *A. niger*, and *Penicillium* spp. Likewise, CuO-NPs synthesized using the stem bark extract of *Syzygium alternifolium* exhibited notable antimicrobial effects against *A. flavus*, *P. chrysogenum*, *A. niger*, and *Trichoderma harzianum* [40]. In a related study, Bukhari et al. [121], synthesized CuO-NPs through the culture supernatant of *Streptomyces* sp. MHM38 and tested their antimicrobial potential against pathogenic *C. albicans*, *Rhizoctonia solani*, *Fusarium solani*, and *A. niger*. Their results indicated that these biosynthesized CuO-NPs effectively inhibited the growth of these pathogenic fungi.

Phyto-mediated CuO-NPs from citrus and tea extracts have shown MICs as low as 50–100 µg/mL against *F. oxysporum* and *A. niger*, disrupting spore germination and hyphal growth [171,172]. These nanoparticles were observed internalizing within fungal hyphae, triggering ROS bursts, mitochondrial membrane collapse, and eventual cell death—confirmed through DCFDA staining and electron microscopy [173]. Furthermore, CuO-NPs inhibit ergosterol biosynthesis, a crucial component of fungal cell membranes. Disruption of ergosterol pathways compromises membrane rigidity and permeability, augmenting the susceptibility of fungi to oxidative damage [174]. Anti-sporulation effects are also notable. Studies using plant pathogenic fungi showed a reduction of up to 80% in spore germination at sub-MIC levels (~50 µg/mL). This attribute is crucial for agricultural use, preventing disease recurrence and fungal spread [175,176]. CuO-NPs’ functional versatility allows them to be integrated into nanocomposites (e.g., with chitosan, cellulose, or polymers) that improve dispersion, reduce toxicity, and enable controlled release. Such composites maintain antifungal efficacy while reducing risk to non-target organisms [177]. Garcia-Marin et al. [117] applied biosynthesized CuO-NPs to treat the pathogenic *C. albicans.* Ultrastructural examination of *C. albicans* showed a substantial accumulation of CuO-NPs both within the cytoplasm and on the cell’s exterior, nanoparticles were also observed in the cell wall. After 24 h of exposure to various CuO-NPs concentrations, these nanoparticles accumulated inside the cells, triggering the generation of ROS. The ROS cause damage to essential cellular components, including DNA, proteins, and lipids, which ultimately results in growth inhibition and cell death [117,178]. The antimicrobial effect of nanoparticles leads to cellular injury by disrupting membrane function—thereby impacting permeability and respiration—and attacking multiple cellular structures. Notably, their capacity to modify proteins containing sulfur and phosphorus allows them to directly interfere with DNA and hinder the organism’s immune responses [117,179].

### 4.3. Antiviral Activity and Mechanisms of Copper/Copper Oxide Nanoparticles

Cu/CuO-NPs have recently gained prominence for their robust antiviral activities, demonstrating broad-spectrum efficacy against both enveloped and non-enveloped viruses. These nanoscale materials not only complement conventional antiviral strategies but also target viruses through multiple mechanisms, reducing the risk of resistance development and enabling faster inactivation rates. According to Ahmadi et al. [79], an extract of *Juglans regia* green husk was utilized to biosynthesize CuO-NPs and the synthesized CuO-NPs were utilized for treating herpes simplex virus type 1 (HSV-1). Moreover, they also used the combination of CuO-NPs and iron oxide nanoparticles (FeNPs) for the treatment of HSV-1. Docking studies confirmed that nanoparticles can bind to HSV-1 glycoproteins, thereby blocking viral entry. The MTT assay showed that the minimum non-toxic dose (MNTD) of CuO-NPs is 100 μg/mL, a concentration that did not demonstrate antiviral effects. However, using a non-toxic dose of iron nanoparticles (FeNPs) at 300 mg/mL together with a cytotoxic dose of CuO-NPs at 300 μg/mL neutralized the toxic effects of CuO-NPs. Treating the virus with this CuO-NPs-FeNPs combination resulted in a 4.5 log_10_ TCID_50_ decrease in HSV-1 viral load, whereas FeNPs alone caused a 3.25 log_10_ TCID_50_ reduction. The study concluded that the combined use of CuO-NPs and FeNPs exhibits antiviral activity against HSV-1 [79]. Liu et al. [34] applied the biologically synthesized CuO-NPs for controlling tobacco mosaic virus (TMV). This research highlighted the green synthesis of CuO-NPs using orange peel extract and their effectiveness in controlling tobacco mosaic virus (TMV) infection both in vitro and in vivo. In vitro exposure of TMV to CuO-NPs at 100 mg/L for 2 h led to noticeable particle fragmentation, which significantly reduced the virus’s infectivity in tobacco plants. Likewise, treatment of *Nicotiana benthamiana* with CuO-NPs successfully suppressed TMV replication and accumulation within the plant. CuO-NP application triggered the upregulation of defense-related genes such as *PR1*, *PR2*, *ERF1*, and *JAZ3*, indicating activation of the plant’s immune response against the virus. Moreover, CuO-NPs at this concentration (100 mg/L) were non-toxic to the host plants and even enhanced physiological traits, including chlorophyll content and both fresh and dry biomass, in comparison to untreated controls. Overall, the study suggested that an optimal CuO-NP concentration of 100 mg/L could directly inactivate viral particles through passivation, enhance plant defense by inducing systemic acquired resistance (SAR), and supply essential nutrients that support plant growth [34].

At the molecular level, CuO-NPs act via several complementary mechanisms: direct disruption of viral envelopes or capsids, oxidation-mediated degradation of viral nucleic acids, and inhibition of viral entry or replication through nanoparticle–protein interactions. For enveloped viruses (e.g., SARS-CoV-2, influenza), CuO-NPs attach to lipid membranes, inducing structural deformation and envelope collapse. This compromises viral integrity and inactivates infectivity. Pieces of research have indicated that copper oxidizes the membrane lipids, creating a cascade of damage that renders the virus non-infectious, often within minutes to hours of contact with coated surfaces. Specifically, a landmark open-access study found that CuNP/paint coatings on stainless steel achieved ~97.8% SARS-CoV-2 inactivation after 30 min, escalating to >99.995% after one hour, significantly outperforming standard painted or uncoated surfaces [180]. Bakhet et al. investigated the virucidal efficacy of coatings made via laser-generated copper nanoparticles (predominantly Cu_2_O/Cu) against infectious bronchitis virus (IBV, a coronavirus) and bovine herpesvirus type 1 (BoHV-1) model viruses for coronavirus and herpesvirus. Their coatings achieved a 98.65–100% reduction in viral titer (measured by TCID_50_ and RT-PCR cycle threshold changes) within short exposure times, with no cytotoxic effects on surrounding cell cultures [181]. Apart from this, Liu et al. [34] used orange peel–synthesized CuO-NPs (100 mg/L) to inhibit tobacco mosaic virus (TMV). After 2 h, TEM imaging showed clear fragmentation of TMV particles, and in planta experiments revealed significant reduction in viral accumulation, confirming capsid disruption and genome inactivation [34]. The same laser-generated Cu_2_O/Cu coatings study elucidated the mechanism of action, explaining how Cu^+^/Cu^2+^ ions generate hydroxyl radicals (·OH), leading to viral membrane disruption, protein oxidation, and genomic damage through Fenton-type reactions [182]. CuO-NPs are integrated into surface coatings, paints, PPE, and filters aimed at interrupting viral transmission via fomites and aerosols [183,184]. These passive interventions maintain long-term antiviral activity without reliance on frequent chemical disinfection, making them ideal for high-touch surfaces, medical environments, and public transit systems. However, while antiviral efficacy is impressive, ensuring human safety remains paramount. Many antiviral evaluations report low cytotoxicity in cell culture, yet in vivo biocompatibility, inhalation risk, and long-term environmental impact require robust investigation.

**Figure 7:**
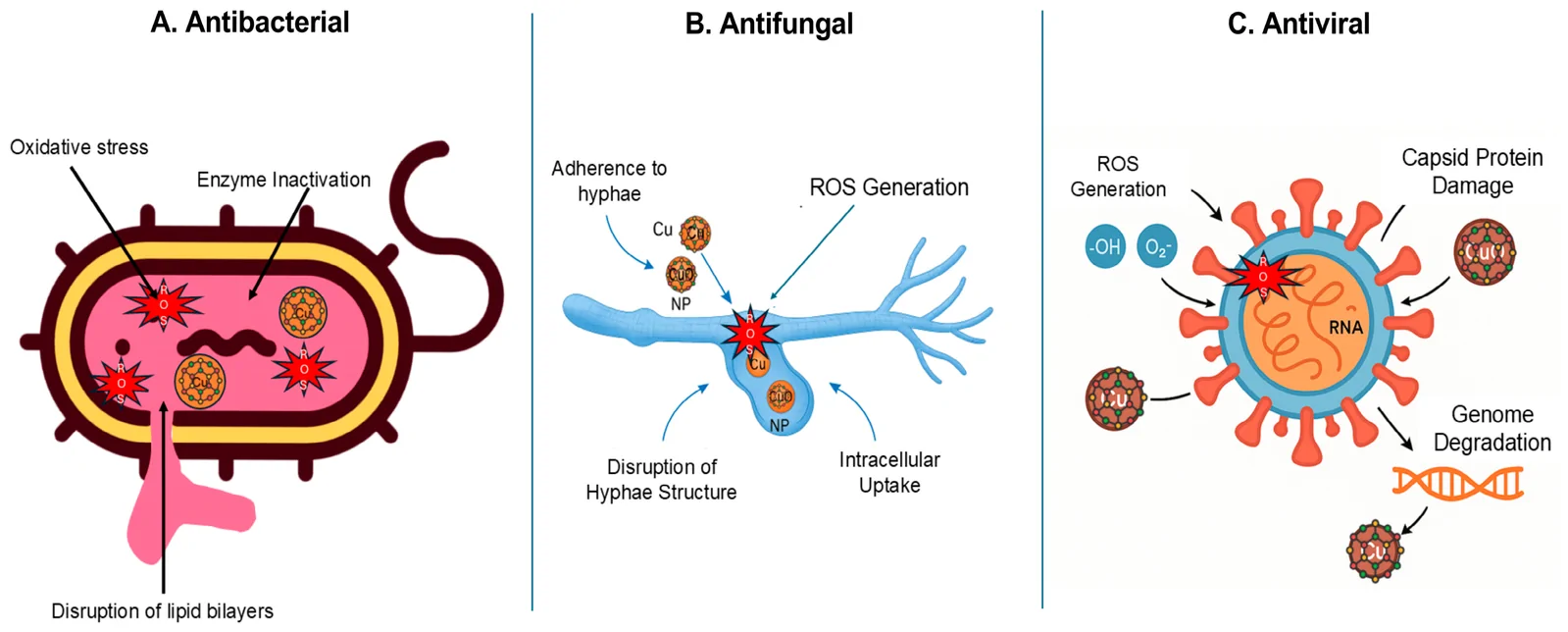
Mechanistic effects of copper and copper oxide nanoparticles on bacteria, fungi, and viruses. (**A**) **Bacteria:** Cu/CuO nanoparticles adhere to bacterial membranes, disrupt lipid bilayers, generate reactive oxygen species (ROS), and inactivate essential iron-sulfur cluster enzymes. These actions result in oxidative stress, cytoplasmic leakage, and eventual bacterial cell lysis. (**B**) **Fungi:** The nanoparticles bind to fungal hyphae and are internalized, where they generate ROS, disrupt hyphal structural integrity, and impair intracellular functions, inhibiting fungal growth and sporulation. (**C**) **Viruses:** Cu/CuO nanoparticles exert antiviral activity through capsid protein denaturation, oxidative cleavage, and degradation of viral nucleic acids (e.g., RNA), thereby blocking replication and rendering the non-infectious virions.

In summary, Cu/CuO-NPs exhibit a range of antimicrobial actions that work together to inhibit microbial growth and viability. These include the production of reactive oxygen species, direct physical interaction with microbial cells, disruption of cell walls and membranes, enzyme inactivation, protein denaturation, and DNA damage. It is important to point out that these mechanisms can differ based on the microorganism involved and the environmental conditions. Gaining insight into how Cu/CuO-NPs exert their antimicrobial effects is vital for developing innovative antimicrobial approaches and enhancing their use across diverse sectors such as healthcare, agriculture, food preservation, and environmental cleanup. Figure 7 shows the proposed antimicrobial mechanisms of Cu/CuO-NPs against bacteria, fungi, and viruses.

### 4.4. Anticancer Activity and Mechanisms of Copper/Copper Oxide Nanoparticles

Cancer is a highly complex and multifactorial disease marked by the uncontrolled proliferation and spread of abnormal cells, resulting from interactions among genetic, environmental, internal, and external factors. It continues to rank among the leading causes of mortality worldwide [185]. In 2026, cancer remains a significant global health burden, with approximately 2.11 million new cases diagnosed annually in the United States alone [186]. Future estimates suggest a substantial rise in incidence, with around 30.5 million new cancer cases and 18.6 million cancer-related deaths expected globally each year by 2050 [187]. Although considerable progress has been made in cancer treatment and medical technologies, the disease remains difficult to manage effectively. Standard therapeutic approaches, including chemotherapy, radiotherapy, and surgical intervention, are often constrained by issues such as poor drug bioavailability, serious side effects, and high treatment costs, all of which can limit their efficacy and accessibility [188,189]. Many anticancer agents exert their effects by disrupting the mitochondrial electron transport chain (ETC) which leads to elevated levels of ROS, damage to cellular structures, and ultimately cell death [190]. Copper and copper oxide nanoparticles (Cu/CuONPs) have emerged as promising candidates in cancer research because of their distinctive quantum-scale properties and large surface-area-to-volume ratio. These characteristics enable them to selectively promote oxidative stress, induce programmed cell death (apoptosis), and suppress tumor cell proliferation [27,191]. Evidence from in vitro and in vivo experiments indicate that Cu/CuONPs can effectively reduce cancer cell viability and inhibit tumor growth [14,116]. Their anticancer activity is primarily associated with enhanced ROS generation, increased oxidative stress, and the selective activation of apoptotic pathways, while causing comparatively low toxicity to normal healthy cells [14,116].

Mahmood et al. [14] reported that CuONPs biosynthesized using the extract of *Annona muricata* demonstrated potent anticancer effects against breast cancer cells. Their findings indicated that the synthesized nanoparticles inhibited cell proliferation by inducing apoptosis-mediated cell death. Similarly, Dolati et al. [116] described the bacterial synthesis of CuO nanoparticles using the culture supernatant of *Bacillus coagulans* and assessed their anticancer potential against breast cancer cells. MTT assay results revealed significant cytotoxic effects of the biosynthesized CuONPs on cancer cells. Molecular analyses demonstrated upregulation of pro-apoptotic and cell cycle regulatory genes, including BAX, P21, CASP3, and CASP9, alongside downregulation of the anti-apoptotic gene BCL2. Furthermore, Annexin V-FITC, propidium iodide staining and ROS analyses confirmed that the biogenic CuONPs induced substantial apoptotic responses compared with the untreated control group [116]. Majeed et al. [41] investigated the green synthesis of CuONPs using *Coriandrum sativum* extract, followed by conjugation with curcumin, and evaluated their effects on oral carcinoma cells. The synthesized curcumin-loaded CuONPs (Cur-CuONPs) exhibited strong cytotoxicity toward KB cancer cells, exhibiting an IC50 of 16.33 µg/mL, while showing considerably lower toxicity toward normal cells (IC50 = 68.26 µg/mL). These findings indicate that Cur-CuONPs possess selective anticancer activity and may serve as promising candidates for targeted drug delivery applications. In another study, Abdollahzadeh et al. [87] synthesized CuONPs through a green approach using walnut shell powder and evaluated their anticancer activity against breast and colon cancer cell lines. MTT assays demonstrated that the synthesized CuONPs exhibited cytotoxic activity against MCF-7, HCT-116, and HEK-293 cells. In addition, Hoechst staining and real-time PCR analyses indicated that the CuO-900 nanoparticles promoted apoptosis through increased expression of the p53 and Bax genes. Based on these findings, the authors concluded that the green-synthesized CuONPs possess considerable anticancer potential against breast and colon cancer cells. Letchumanan et al. [81] also reported the green synthesis of CuONPs using *Camellia sinensis* extract and examined their effects on human breast (MCF-7) and colon (HT-29) cancer cell lines. Microscopic observations and fluorescence imaging conducted after 24 h of treatment revealed a substantial reduction in cell viability in both cancer cell lines. Viability in HT-29 cells decreased from 98.50% in untreated controls to 28.45% following treatment, whereas MCF-7 cell viability declined from 97.50% to 35.75%. The pronounced reduction in viable cells suggests that the nanoparticles exert strong cytotoxic effects, likely through membrane disruption and the induction of cell death. Overall, the study demonstrated that biosynthesized CuONPs possess significant anticancer activity against both colon and breast cancer cells, supporting their potential application as therapeutic agents [81].

Copper and copper oxide nanoparticles have gained significant interest as potential anticancer therapeutics due to their capacity to selectively target and eliminate cancer cells through multiple biological mechanisms. One of their principal anticancer actions involves the generation of ROS through the redox activity of copper ions. Consequently, oxidative stress triggers lipid peroxidation, protein damage, and DNA degradation within tumor cells. Elevated ROS levels also impair mitochondrial membrane stability, thereby promoting cytochrome c release and the activation of caspase-mediated apoptotic pathways, ultimately promoting programmed cell death. In addition to inducing apoptosis, Cu/CuONPs can inhibit tumor growth by arresting the cell cycle at specific phases, thereby preventing cellular proliferation. These nanoparticles have also been reported to stimulate autophagic processes and inhibit tumor angiogenesis through disruption of vascular endothelial growth factor associated signaling cascades. Furthermore, their distinctive physicochemical characteristics make them suitable for use in photothermal and photodynamic therapies, where externally applied light stimulates localized heat generation or ROS production, enhancing cancer cell destruction. The selective cytotoxicity of Cu/CuONPs toward malignant cells is largely associated with the elevated oxidative stress levels and increased metabolic demands characteristic of cancer cells, rendering them more susceptible to nanoparticle-induced injury than normal cells. Collectively, these diverse mechanisms underscore the significant potential of copper-based nanomaterials as promising candidates for the development of advanced cancer therapies [41,81,192,193].

**Figure 8:**
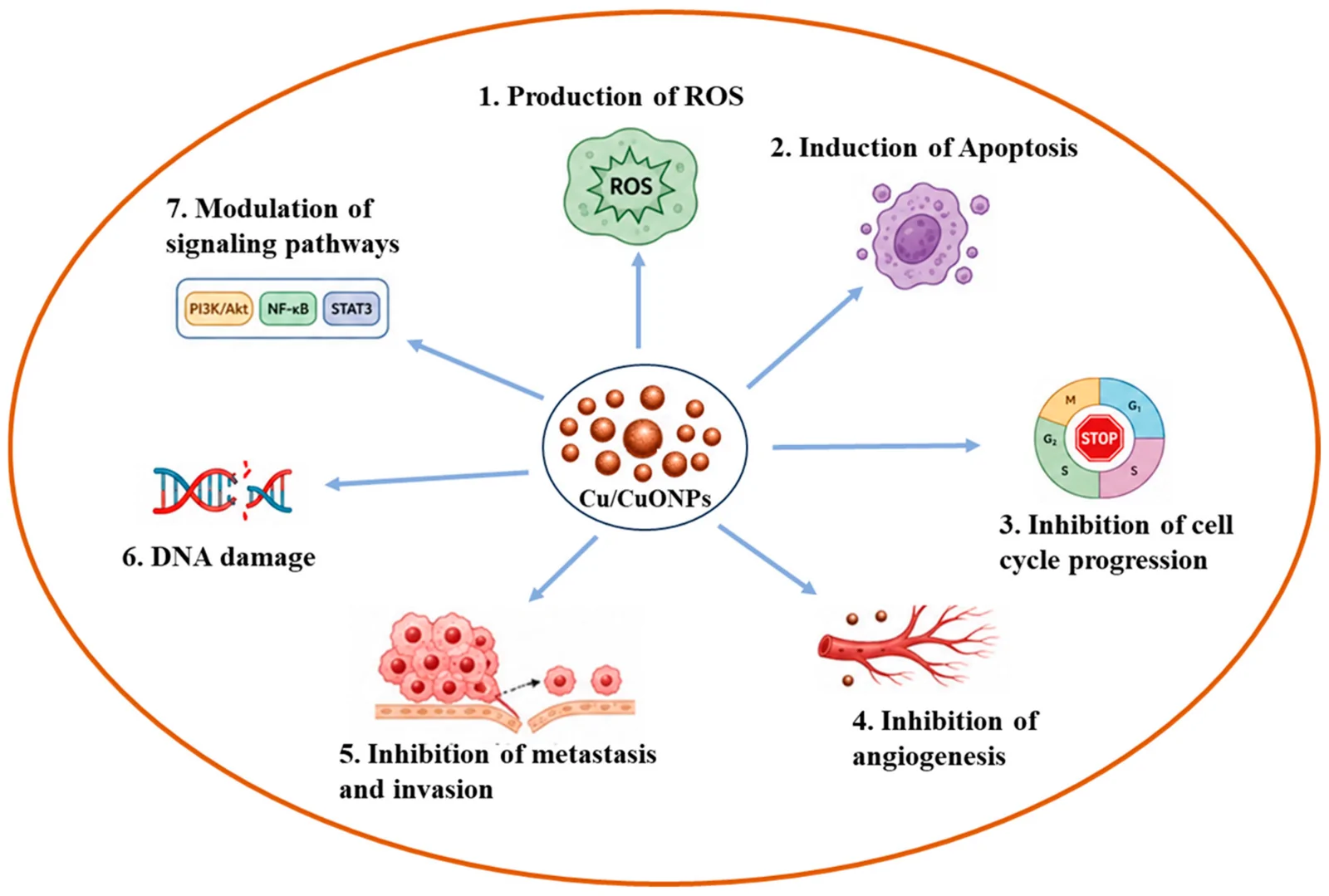
Proposed anticancer mechanisms of biogenic Cu/CuO-NPs.

Majeed et al. [41] suggested that biosynthesized CuONPs exert their anticancer effects against oral carcinoma cells through several interconnected mechanisms. These include the induction of morphological changes and membrane blebbing, increased generation of ROS, and DNA damage. Furthermore, the nanoparticles were found to suppress the expression of the anti-apoptotic Bcl-2 gene, while upregulating the expression of key pro-apoptotic genes, such as p53, Bax, caspase-3, caspase-8, and caspase-9, thereby promoting apoptosis in cancer cells. Likewise, Letchumanan et al. [81] demonstrated that CuONPs synthesized using *Camellia sinensis* extract possess significant anticancer activity against human colon and breast cancer cell lines. Their findings revealed that the nanoparticles induced dose-dependent cytotoxicity and apoptosis, with oxidative stress playing a central role in their mechanism of action. These results highlight the therapeutic potential of biosynthesized CuONPs as effective agents for targeting and eliminating cancer cells. In a related investigation, Pahlevanhosseini et al. [73] employed *Mentha longifolia* extract for the green synthesis of silver–copper oxide nanoparticles (Ag-CuO-NPs) and evaluated their anticancer mechanisms against gastric and breast cancer cells. Flow cytometric analysis revealed that treatment with the synthesized nanoparticles induced substantial levels of late apoptosis, reaching 24.4% in MCF-7 cells and 20.8% in AGS cells, while necrosis rates were 6.63% and 6.88%, respectively. These effects were markedly greater than those observed in cells treated with the plant extract alone. Gene expression analysis demonstrated increased expression of the pro-apoptotic genes such as Bax, Caspase-3, and P53, alongside reduced expression of the anti-apoptotic gene Bcl-2 in nanoparticle-treated cells. Additionally, significant elevations in both ROS and nitric oxide levels were detected following nanoparticle exposure. The study concluded that *M. longifolia*-derived nanoparticles effectively promote apoptosis through oxidative stress-mediated pathways and may serve as a promising nanotherapeutic approach for the treatment of breast and gastric cancers. In conclusion, Cu/CuONPs exhibit a broad spectrum of anticancer activities that contribute to their ability to inhibit tumor progression and promote cancer cell death. Their mode of actions includes the generation of ROS and subsequent oxidative stress, induction of apoptosis through mitochondrial dysfunction and caspase activation, disruption of cell cycle progression, inhibition of angiogenesis, suppression of cancer cell invasion and metastasis, induction of DNA damage, and regulation of various cellular signaling pathways. Collectively, these multifaceted effects highlight the therapeutic potential of biogenic Cu/CuO-NPs as effective anticancer agents. The proposed mechanisms underlying their anticancer activity are illustrated in Figure 8.

## 5. Conclusions, Challenges and Prospective

A review centered on the biosynthesis, characterization and antimicrobial and anticancer potential of Cu/CuO-NPs is valuable, as it emphasizes an emerging strategy for developing novel antimicrobial and anticancer solutions amid rising drug resistance. This review provides an overview of various green synthesis strategies for Cu/CuO-NPs, with emphasis on their physicochemical characteristics and their potential antimicrobial and anticancer activities against diverse pathogens and cancer cell lines. Furthermore, it may highlight their broader biomedical prospects, including applications as antibacterial, antifungal, antiviral and even anticancer agents.

Biological synthesis of Cu/CuO-NPs is often considered more sustainable, economical, and potentially biocompatible compared to conventional chemical routes. In contrast, chemical synthesis provides better precision in tailoring particle size and morphology, whereas green synthesis typically produces nanoparticles with broader size distributions. Both synthesis strategies can produce Cu/CuO-NPs exhibiting notable antimicrobial and anticancer effects; however, their properties and performance are strongly influenced by the selected synthesis route and the specific application for which they are intended [27,194]. Importantly, Cu/CuO-NPs produced through green synthesis generally demonstrate enhanced biological activity and improved biocompatibility compared with those generated by conventional chemical methods [195,196]. Furthermore, these nanoparticles generally exhibit reduced cytotoxicity toward mammalian cells, supporting their potential biomedical applications; however, their toxicity remains concentration-dependent, as higher doses can compromise cellular viability [196,197].

Both infectious diseases and cancers are major public health concerns that cause morbidity and mortality across the globe. Cu/CuO-NPs showed effective antimicrobial activities against various pathogenic microbes, including bacteria, fungi, viruses and demonstrated various antimicrobial mechanisms. CuO-NPs hold significant promise in the field of nanomedicine due to their strong antibacterial, antifungal, antiviral, and anticancer capabilities. The antimicrobial and anticancer effectiveness of Cu/CuO-NPs is influenced by various factors, including their size, shape, surface charge, concentration, the nature of the capping agents used, and the specific targeted microorganisms or cancer cells. Common mechanisms by which these nanoparticles exert their action include disruption of the cell wall and membrane, penetration into cells, generation of ROS, interference with DNA integrity, and inhibition of protein synthesis. This makes Cu/CuO-NPs a promising candidate for the development of new antimicrobial and anticancer agents that can combat pathogenic bacteria, fungi and viruses as well as cancer cells. While biologically synthesized Cu/CuO-NPs offer significant efficacy against pathogenic microbes and cancer cells, challenges remain in their widespread application. These include potential toxicity, availability, regulatory hurdles, and difficulties in controlling the size, shape, and stability of the synthesized NPs.

Despite significant advancements in the biogenic synthesis of medically important metal-based nanoparticles, several challenges remain unresolved. One of the main difficulties in biological nanoparticle production lies in achieving precise control over size distribution, shape, and surface chemistry. In this regard, chemical and physical synthesis methods often offer better control over these characteristics than biological approaches. This problem could be overcome by optimizing the synthesis condition in biological method, like optimum temperature, pH, extract and precursor concentration, etc. Such optimization is a common approach in nanoparticle synthesis, where these parameters significantly influence the outcome. By carefully adjusting these factors, it is often possible to improve the quality, stability, yield, or other desired characteristics like shape and size of the synthesized nanoparticles. Biological activity of biosynthesized Cu/CuO nanoparticles is influenced by their physicochemical properties, particularly particle size and morphology. In general, smaller nanoparticles tend to exhibit enhanced antimicrobial and anticancer activity because of their greater surface reactivity and improved interaction with biological systems. However, biological performance is also affected by other factors, including chemical composition, oxidation state, surface chemistry, crystallinity, and experimental conditions. Similarly, nanoparticle morphology may affect surface reactivity and the mode of interaction with biological membranes, thereby influencing cellular internalization and biological responses. However, the relationship between nanoparticle size, shape, and biological activity is not straightforward. Differences in precursor chemistry, oxidation state, crystallinity, surface functionalization by biological capping agents, colloidal stability, dosage, exposure time, and experimental protocols can all significantly affect the reported outcomes. Comprehensive physicochemical characterization, together with standardized biological evaluation, is essential for accurately correlating nanoparticle properties with their antimicrobial and anticancer performance.

While Cu/CuO-NPs show promise for biomedical applications, their potential toxicity to both vertebrates and invertebrates raises important safety concerns regarding their use in diagnostics and therapy. Cu/CuO-NPs can induce oxidative stress by generating excessive levels of ROS, which may lead to damage in DNA and cellular organelles. Key factors contributing to their toxicity, both in vitro and in vivo, include particle size, surface charge, and their dissolution behavior. Therefore, ensuring biocompatibility and minimizing toxicity are essential criteria when selecting nanoparticles for clinical research. Nanoparticles, when used in medical applications, come into direct contact with living tissues and cells, making their compatibility with the body a primary concern. Furthermore, the potential for toxicity, even with promising drug delivery capabilities, necessitates careful evaluation. Conjugating antimicrobial or anticancer drugs or ligands with biogenic Cu/CuO-NPs can reduce toxicity and improve biocompatibility in certain contexts. This approach allows for targeted drug delivery, minimizing exposure to healthy cells and reducing overall toxicity. Additionally, surface modifications with biocompatible materials can enhance the nanoparticle’s compatibility with biological systems. Studies indicate that polymer-functionalized nanoparticles often demonstrate superior performance compared to their individual components. The conjugation with polymers enhances nanoparticle stability, enables controlled copper ion release, and provides synergistic interactions that improve cell membrane penetration and disruption, thereby increasing microbial eradication efficiency [198]. Polymer-coated Cu/CuO-NPs facilitate sustained activity by maintaining a steady ion release and offering targeted action, while the polymer matrix mitigates copper ion toxicity, improves bioavailability through surface modification, and prolongs circulation time. The polymeric coating helps inhibit nanoparticle aggregation, thereby improving their stability and functional performance in both biomedical and environmental applications. In addition, surface functionalization with drugs, ligands, or other bioactive compounds can further enhance the biocompatibility and therapeutic effectiveness of Cu/CuO-NPs. This strategy combines multiple mechanisms of action, amplifies oxidative stress, and reduces the risk of drug resistance by simultaneously targeting several cellular pathways [199]. Developing Cu/CuO-NPs within metal–organic frameworks (MOFs) represent a promising and effective strategy for creating novel antimicrobial and anticancer agents, owing to the synergistic effect of the highly porous MOF structure and the antimicrobial and anticancer properties of copper. Copper ions (Cu^2+^/Cu^+^) can oxidize bacterial proteins and lipids, causing cell death. The porous and highly functionalized structure of MOFs allows them to act as efficient carriers, providing a large surface area for Cu/CuO-NPs and releasing copper ions in a controlled manner. The combination of copper and the MOF structure create a synergistic effect, enhancing the overall antimicrobial as well as anticancer potency compared to using either component alone. These nanocomposites can disrupt cells through mechanisms like oxidative stress, release of copper ions, and physical damage to cell walls, demonstrating significant potential for applications such as water purification and wound healing [200,201,202]. Combining drugs, ligands, bioactive polymers, or MOFs with biogenic Cu/CuO-NPs could be a promising strategy for creating safe and effective antimicrobial agents against pathogenic bacteria, fungi, and viruses as well as anticancer agents against various cancer cells. Such conjugation enables targeted delivery, thereby limiting exposure to healthy cells and lowering overall toxicity. We hope that this review will encourage additional research in this dynamic and medically significant field.

## Data Availability

No new data were created or analyzed in this study. Data sharing is not applicable to this article.
